# Efficacy of different types of transcranial magnetic stimulation on post-stroke aphasia patients: a network meta-analysis

**DOI:** 10.3389/fneur.2025.1597504

**Published:** 2025-07-28

**Authors:** Pei Li, Renyan Xiao, Meng Gong, Peng Jia, Song Jin

**Affiliations:** ^1^School of Health Preservation and Rehabilitation, Chengdu University of Traditional Chinese Medicine, Chengdu, China; ^2^School of Acupuncture and Tuina, Chengdu University of Traditional Chinese Medicine, Chengdu, China; ^3^Department of Rehabilitation, Hospital of Chengdu University of Traditional Chinese Medicine, Chengdu, China

**Keywords:** transcranial magnetic stimulation, rTMS, aphasia, stroke, TBS, network meta analysis

## Abstract

**Objective:**

To evaluate the comparative efficacy of repetitive transcranial magnetic stimulation (rTMS) for post-stroke aphasia through a network meta-analysis (NMA).

**Methods:**

We systematically searched four international databases (PubMed, Cochrane Library, Web of Science, and Embase) and three Chinese databases (CNKI, Wanfang Data, and VIP) from inception through October 2024. Two reviewers independently performed literature screening, data extraction, and quality assessment. Discrepancies are resolved through third-party adjudication. Network meta-analysis was conducted using Stata 17.0.

**Results:**

Thirty-eight randomized controlled trials (RCTs, *n* = 1,982) were included, and the NMA results showed that (1) Low-frequency rTMS (LF-rTMS) in combination with High-frequency rTMS (HF-rTMS) (LF-rTMS & HF-rTMS) showed well performance in all indicators (2); intermittent theta-burst stimulation (iTBS) exhibited well benefits for comprehension and repetition domains (3); Continuous theta-burst stimulation (cTBS) showed superior efficacy in naming (4). Stimulation of the pars opercularis (POp) was superior to the pars triangularis (PTr) within the inferior frontal gyrus (IFG) (5). Anatomical landmarks (AL) was the predominant targeting method.

**Conclusion:**

LF-TMS & HF-TMS is the most effective intervention for clinical treatment of post-stroke aphasia. When targeting the IFG, POp is the optimal stimulation site. Regarding targeting methods, the 10–20 EEG system currently has the strongest evidence base.

## 1 Introduction

Aphasia, an acquired neurogenic language disorder, arises from damage to neuroanatomical substrates primarily located in the dominant (left) hemisphere. This condition is characterized by impaired verbal expression, written communication, or auditory comprehension. While multiple etiologies, including traumatic brain injury, neoplastic processes, and neurodegenerative conditions, may cause it, cerebrovascular accidents-particularly ischemic stroke in the left middle cerebral artery (MCA) territory-represent the predominant etiology. Epidemiological studies indicate that stroke accounts for approximately 33% of incident aphasia cases annually. Clinically, post-stroke aphasia (PSA) invariably manifests as social communication deficits, diminished quality of life, and occupational disability, regardless of severity (1). Contemporary therapeutic approaches encompass pharmacotherapy, speech-language therapy (SLT), melodic intonation therapy, and non-invasive brain stimulation (NIBS) techniques, among others (2).

The operating principle of TMS is grounded in Faraday’s law of electromagnetic induction. When a coil positioned on the scalp carries a high-intensity, pulsed electric current, it generates a brief, focused, time-varying magnetic field. This magnetic field penetrates the skull almost unimpeded, inducing an electric field within the target region of the cerebral cortex (3). When reaching sufficient strength, this induced electric field causes depolarization of cortical neurons, triggering action potentials (4).

The current study suggests that rTMS (mainly low-frequency) applied to homologous language areas in the right cerebral hemisphere (e.g., the dorsum of the right inferior frontal gyrus or the temporoparietal region) can promote functional recovery within the left hemisphere language network. This effect is hypothesized to occur through the inhibition of overactive compensatory activity in the right hemisphere or the reduction of its pathological transhemispheric inhibition (5). When administered as a stand-alone intervention, rTMS has been shown to enhance language (6–9) and cognitive functions in PSA (10). However, a more common therapeutic strategy involves its use as an adjunct to conventional speech-language therapy (SLT), wherein rTMS is applied prior to or concurrently with SLT sessions. This combined approach aims to transiently optimize cortical excitability states via rTMS, thereby potentiating the neuroplastic effects induced by subsequent language training (11). Research shows that rTMS-assisted speech therapy typically yields superior outcomes than speech therapy alone (12). The therapeutic mechanism involves generating pulsed magnetic fields that alter cortical neuronal membrane potentials, thereby inducing currents capable of modulating cerebral metabolism and neuroelectrophysiological activity. Under physiological conditions, interhemispheric homeostasis is maintained through corpus callosum-mediated reciprocal inhibition between cerebral hemispheres. Following brain injury, this equilibrium is disrupted. Specifically, reduced inhibition from the dominant hemisphere leads to a relative increase in excitability within the non-dominant hemisphere. rTMS protocols are designed to either excite the lesioned (dominant) hemisphere or inhibit the non-lesioned (non-dominant) hemisphere, thereby restoring interhemispheric balance and promoting functional recovery in aphasia patients (13).

The therapeutic efficacy of TMS is closely related to the frequency of stimulation, the stimulation site, the dosage, and the method of localization (14, 15). Based on stimulation frequency, rTMS is broadly categorized into LF-rTMS and HF-rTMS. TBS, an optimized rTMS paradigm, operates in two distinct modes: iTBS and cTBS (16). Both types of rTMS have been investigated for the treatment of PSA, with strategies also exploring bilateral stimulation protocols and multi-target approaches.

NMA facilitates simultaneous direct and indirect comparisons of multiple rehabilitation interventions, enabling the evaluation of clinical outcomes across two or more treatment modalities. However, current comparative effectiveness research on repetitive transcranial magnetic stimulation (rTMS) protocols for post-stroke aphasia (PSA) remains limited, with most studies focusing on isolated interventions. While one meta-analysis (17) incorporated both rTMS and transcranial direct current stimulation (tDCS), it provided insufficient detail on rTMS treatments specifically and lacked a detailed analysis of key TMS parameters. Furthermore, although prior meta-analyses (14, 15) investigated optimal parameters for rTMS in aphasia, they did not perform a detailed meta-analysis of stimulation sites or targeting methods. Also, published systematic reviews of rTMS for post-stroke aphasia show low methodological quality, and evidence of its effectiveness remains inconclusive (18). Therefore, to address these gaps, this study employs an NMA approach to systematically evaluate the efficacy of rTMS techniques differing in frequency, stimulation site, and targeting method. By comparing the intervention effects of distinct rTMS paradigms, this research aims to establish an evidence-based framework to guide optimal rTMS protocol parameter selection for PSA rehabilitation.

## 2 Methods

We strictly followed the PRISMA extension statement for reporting systematic reviews incorporating NMA of health care interventions (PRISMA-NMA). The study protocol was registered in PROSPERO (CRD42024625471). Ethical approval is not required because the information used in this study is obtained from published randomized controlled trials (RCTs).

### 2.1 Inclusion criteria

Participants: Diagnosed with post-stroke aphasia using standardized assessment scales; Intervention: Repetitive transcranial magnetic stimulation (rTMS) combined with routine speech rehabilitation training; Comparison: Sham stimulation or placebo therapy; Outcomes: The primary outcomes encompassed changes in the global severity of aphasia and performance across speech subdomains. Speech subdomains assessed included spontaneous speech, comprehension, repetition, and naming. Aphasia severity was assessed using scores derived from the Aphasia Quotient (AQ) of the Western Aphasia Battery (WAB) or other scales [The Aachener Aphasie Test (AAT), the Boston Diagnostic Aphasia Examination (BDAE), the Concise Chinese Aphasia Test (CCAT), and Chinese Rehabilitation Research Center Aphasia Examination (CRRCAE), Aphasia Battery of Chinese (ABC)]; Study type: Articles published in Chinese or English; Randomized controlled trial with human subjects.

### 2.2 Exclusion criteria

Studies recruiting ineligible participants, such as studies recruiting only healthy volunteers or pediatric populations, are excluded from the study; use of non-relevant interventions, such as invasive interventions (e.g., deep brain stimulation) and or incorporating significant co-interventions; inappropriate control group, studies featuring control groups that were not adequately matched to the intervention group regarding key demographic (e.g., age, sex) or clinical characteristics (e.g., baseline aphasia severity); studies that failed to report the primary outcome measures specified in this review, or where the reported outcome data were inaccessible (e.g., not reported, presented only graphically without raw/numerical data), inconvertible using standard statistical methods, or contained significant errors.

### 2.3 Search strategy

A comprehensive literature search was conducted across four international databases (PubMed, Cochrane Library, Web of Science, and Embase) and three Chinese databases [China National Knowledge Infrastructure (CNKI), Wanfang, and VIP] from inception until October 2024.

The search strategy utilized a combination of Medical Subject Headings (MeSH)/Emtree terms and relevant free-text keywords. Key search terms included, but were not limited to: “Aphasia,” “Stroke,” “Transcranial Magnetic Stimulation,” and “Randomized Controlled Trial.” Equivalent Chinese terms were employed for searches within the Chinese databases. The search strategy was tailored to the specific syntax and indexing features of each database.

### 2.4 Literature screening and data extraction

Two researchers (PL and RX) independently applied the inclusion and exclusion criteria to conduct a two-stage screening process of titles and abstracts, followed by full-text assessment. Data extraction was also performed independently by both researchers, with subsequent cross-checking. Disagreements regarding study eligibility or data extraction were resolved through discussion with a third researcher (MG).

### 2.5 Quality assessment

Two researchers (PL and RX) independently assessed the risk of bias according to the Cochrane Risk of Bias tool version 2 (ROB2); assessments were performed across the following domains: (1) Randomization process; (2) Deviations from the intended interventions; (3) Missing outcome data; (4) Measurement of the outcome; and (5) Selection of the reported result. Each domain was judged as having “Low risk,” “Some concerns,” or “High risk” of bias. Any discrepancies between the independent assessments were resolved through discussion with a third researcher (MG).

### 2.6 Statistical analysis

Statistical analyses were performed using Stata 17.0 software. As all outcome measures in this study were continuous variables, treatment effects were expressed as standardized mean differences (SMDs) with 95% confidence intervals (CIs).

First, the network relationships for each outcome were visualized using appropriate network commands. If closed loops were present within the network, global inconsistency was evaluated using the design-by-treatment interaction model. A consistency model was employed for analysis if the inconsistency test yielded *p* > 0.05. The node-splitting approach and loop-specific inconsistency tests were applied to examine local inconsistency. The relative ranking probabilities of interventions were estimated and summarized using the surface under the cumulative ranking curve (SUCRA). A higher SUCRA value indicates a more favorable ranking. For interventions with fewer than three contributing studies, SUCRA values were considered potentially unstable. Sensitivity analyses were conducted by excluding such interventions to assess the robustness of the primary findings. Potential publication bias and small-study effects were evaluated using funnel plots.

Additionally, stimulation site and targeting method were pre-specified as secondary outcomes. Subgroup analyses or meta-regression (as applicable) were performed on the included studies categorized by these factors, following the analytical framework described above.

## 3 Results

### 3.1 Study selection

**Figure 1:**
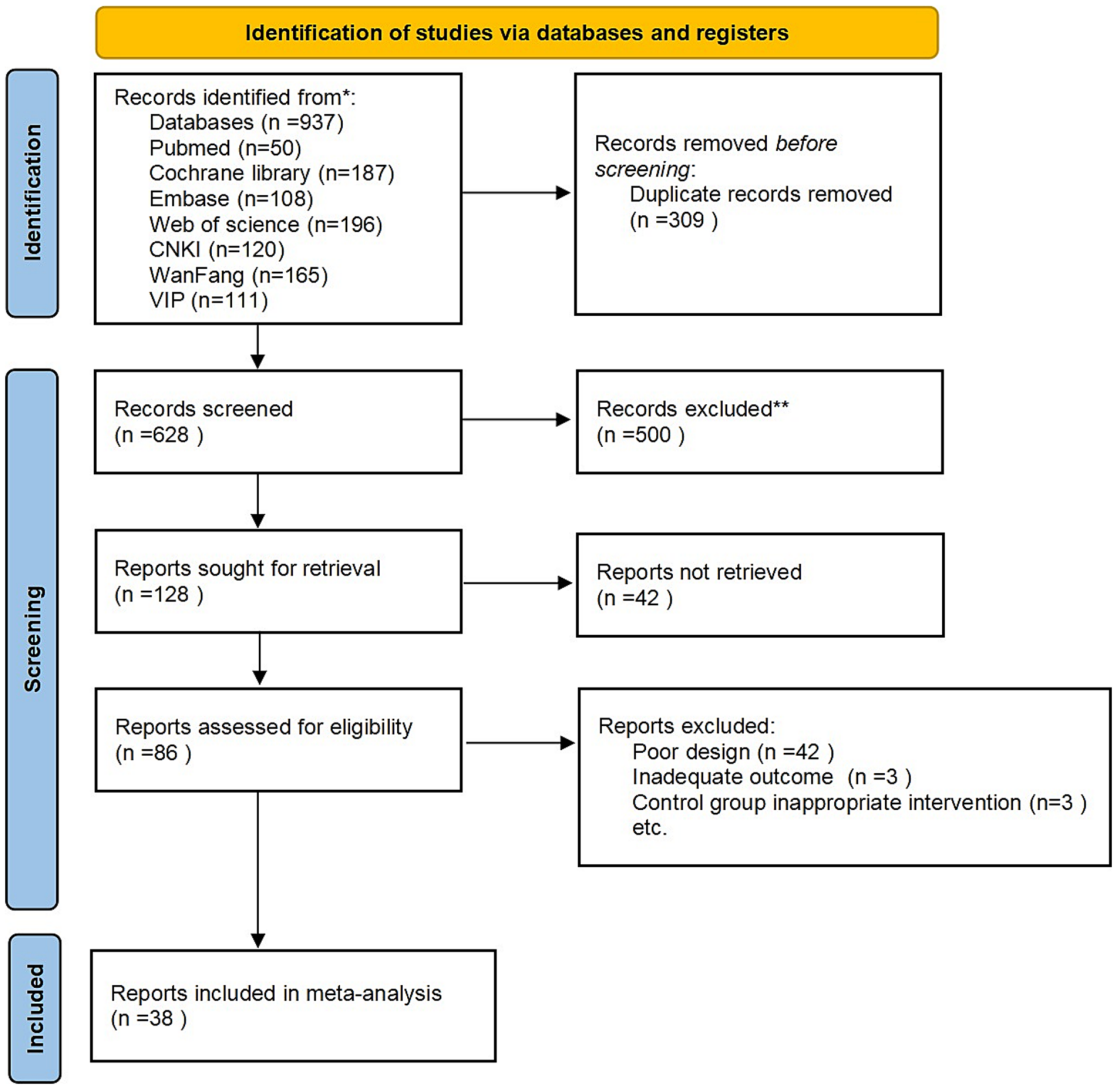
Flow chart of the study identification, screening, eligibility assessment, and inclusion processes.

A total of 937 records were screened, of which 309 were duplicates. Based on the inclusion and exclusion criteria, 38 articles were finally included in this NMA. The flowchart of the literature screening and inclusion process is shown in Figure 1.

### 3.2 Study characteristics

Thirty-eight randomized controlled trials (19–56) with a total of 1982 patients were included in this NMA, details of which are shown in Table 1. The mean age of the patients ranged from 45 to 70. The majority of the patients were in the subacute and chronic phases of stroke, with only five studies (31, 50, 51, 54, 55) including patients in the acute phase. Female participants comprised approximately 40% of the total sample. Thirty-four were recorded in Chinese, 2 in Polish, 1 in Persian, and 1 in German as the first language. Except for one article which did not specify the stimulation site, the remaining 37 were recorded, of which one article stimulated the superior frontal gyrus (SFG), two articles stimulated the superior temporal gyrus (STG), the inferior frontal gyrus (IFG) was documented in 34 papers, including 11 papers stimulating the pars triangularis (PTr) and 21 papers stimulating the pars opercularis (POp). Localization methods were reported in 26 studies. Among these, seven studies utilized a neuronavigational system (NN).

**Table 1:** Summary characteristics of studies included in the review.

Study	Treatment method	Number of participants	Sex (male/female)	Mean age (years, SD)	Time post-onset mean (SD)	Duration of each session	Intervention time	Outcome indicators	First Language	Site of stimulation	Targeting method
Hu XY 2018	H-TMS	10	7/3	46.5 (12.1)	7.1 (2.7) (m)	10 min	10 d	WAB	Chinese	IFG-POp	AL
	L-TMS	10	6/4	48.5 (11.2)	7.5 (3.2) (m)						
	Sham	10	5/5	50.7 (10.4)	6.8 (2.3) (m)						
	Placebo	10	6/4	47.3 (9.8)	7.7 (3.4) (m)						
Hu XY 2023	H-TMS	18	13/7	54.50 (11.20)	32.33 (10.54) (d)	NM	10 (2 w)	WAB	Chinese	IFG-POp	AL
	L-TMS	18	15/5	52.72 (11.80)	37.00 (11.50) (d)						
	Sham	17	14/6	58.58 (9.35)	36.47 (12.15) (d)						
	Placebo	18	14/6	56.83 (11.22)	35.39 (11.40) (d)						
Ren J 2023	iTBS	15	10/5	52.53 (11.84)	2.57 (2.50) (m)	3.2 min	15 (3 w)	WAB	Chinese	SFG	NN
	cTBS	15	13/2	53.13 (12.19)	2.65 (1.38) (m)						
	Sham	14	9/5	51.93 (12.80)	1.79 (1.52) (m)						
Yuan H 2024	H-TMS & L-TMS	15	11/4	69.87 (6.51)	<2 w	20 min	10 d	WAB	Chinese	IFG-POp	NA
	L-TMS	15	10/5	69.47 (6.45)							
	Placebo	15	10/5	68.20 (7.50)							
Bai G 2020	2L-TMS	10	13/17	45.3 (6.8)	3.0 (1.5) (m)	20 min	20 (4 w)	WAB	Chinese	IFG	AL
	L-TMS	10									
	Sham	10									
Haghighi M 2017	L-TMS	6	3/3	61.67 (7.06)	4–8 w	20 min	10 (2 w)	WAB	Farsi	IFG-POp	NA
	Sham	6	2/4	60.5 (11.85)							
Bai G 2022	L-TMS	30	17/13	63.47 (7.81)	3.27 (1.50) (m)	NM	20 (4 w)	WAB	Chinese	IFG-POp	AL
	Sham	28	14/16 (detachment)	59.91 (8.58)	3.75 (1.67) (m)						
Zhou HY 2021	L-TMS	53	30/23	61.25 (8.41)	9.35 (3.27) (w)	NM	20 (4 w)	WAB	Chinese	IFG-POp	AL
	Placebo	53	28/25	59.87 (7.64)	8.91 (2.36) (w)						
You L 2021	L-TMS	22	16/6	59.3 (12.4)	51.3 (14.4) (d)	NM	18 (3 w)	WAB	Chinese	IFG-POp	AL
	Placebo	22	15/7	57.1 (10.7)	48.8 (17.6) (d)						
Ju YZ 2020	L-TMS	20	13/7	68.60 (7.78)	26.50 (12.51) (d)	NM	10 (2 w)	WAB	Chinese	IFG-POp	NA
	Sham	20	14/6	67.80 (7.32)	25.80 (11.77) (d)						
Rubi-Fessen I 2015	L-TMS	15	NM	67.9 (8.12)	41.47 (21.51) (d)	20 min	2 w	ATT	German	IFG-PTr	NN
	Sham	15	NM	69.60 (6.67)	48.73 (21.57) (d)						
Tsai PY 2014	L-TMS	33	24/9	62.3 (12.1)	17.8 (7.2) (w)	10 min	10 d	CCAT	Chinese	IFG-PTr	NN
	Sham	23	17/6	62.8 (14.5)	18.3 (8.2) (w)						
Lin B 2022	L-TMS	17	11/6	54.71 (12.03)	9.41 (7.27) (w)	15 min	10 d	CCAT	Chinese	IFG-PTr	NN
	Sham	16	11/5	62.94 (14.59)	12.63 (12.90) (w)						
Chou T 2021	iTBS	29	15/14	62.7 (12.7)	17.6 (20.8) (w)	190 s	10 (2 w)	CCAT	Chinese	IFG-PTr	NN
	L-TMS	27	19/8	56.9 (13.2)	13.2 (21) (w)	20 min					
	Sham	29	20/9	61.6 (14.7)	16.5 (24.6) (w)	NM					
Mao JN 2021	H-TMS	33	19/14	61.13 (1.24)	4.43 (0.61) (d)	NM	20 (4 w)	ABC	Chinese	IFG-POp	NA
	Sham	31	18/13	60.52 (1.52)	5.03 (0.52) (d)						
Gu HP 2019	L-TMS	50	28/22	65.69 (7.21)	1.82 (0.64) (m)	NM	20 (4 w)	ABC	Chinese	STG	NA
	Sham	50	29/21	67.30 (6.51)	1.95 (0.72) (m)						
Ren CL 2019	L-TMS	13	7/6	62.46 (10.95)	50.58 (23.80) (d)	20 min	15 (3 w)	WAB	Chinese	IFG-PTr	AL
	Sham	15	9/6	63.60 (16.71)	61.20 (22.66) (d)						
Li W 2019	L-TMS	15	11/4	57.00 (1.24)	NM	NM	10 (2 w)	WAB	Chinese	IFG-PTr	AL
	Sham	15	9/6	47.07 (1.37)	NM						
Wang CP 2014	L-TMS	15	13/2	62.1 (12.7)	15.7 (8.5) (w)	20 min	10 d	CCAT	Chinese	IFG-PTr	NN
	Sham	15	13/2	60.4 (11.9)	16.1 (7.3) (w)						
Yoon TH 2015	L-TMS	10	8/2	60.46 (9.64)	6.80 (2.39) (m)	20 min	20 (4 w)	WAB	Chinese	IFG	AL
	Placebo	10	7/3	61.13 (8.73)	5.20 (2.67) (m)						
Lin ZH 2017	L-TMS	13	9/6 (detachment)	65.3 (5.6)	47.5 (7.4) (d)	20 min	15 (3 w)	WAB	Chinese	IFG-POp	NN
	Sham	13	7/8 (detachment)	68.3 (5.8)	51.0 (9.6) (d)						
Shen Y 2016	L-TMS	20	11/9	60.2 (10.5)	50.7 (16.3) (d)	NM	15 (3 w)	WAB	Chinese	IFG-POp	AL
	Placebo	20	8/12	57.5 (11.9)	45.1 (18.8) (d)						
Feng Y 2013	L-TMS	12	7/5	NM	NM	20 min	10 (2 w)	ABC	Chinese	IFG-PTr	NN
	Placebo	12	6/6	NM	NM						
Ren CL 2018	L-TMS	6	3/3	66.4	2.2 (0.5) m	20 min	15 (3 w)	WAB	Chinese	STG	AL
	Sham	6	4/2	64	2.7 (0.6) m						
Fang Y 2018	H-TMS & L-TMS	48	28/20	64.3 (15.7)	10.3 (3.7) (d)	20 min	20 (4 w)	WAB	Chinese	IFG-POp	NA
	Placebo	52	30/22	63.5 (16.5)	10.7 (3.5) (d)						
Lai JH 2019	c-TBS	11	7/4	62.45 (11.01)	2 w–3 m	NM	20 (4 w)	WAB	Chinese	IFG-POp	NA
	L-TMS	12	8/4	61.92 (8.66)							
	Placebo	12	8/4	60.58 (9.98)							
Hui P 2020	L-TMS	40	26/14	59.79 (5.85)	10.39 (2.83) (d)	20 min	20 (4 w)	WAB	Chinese	IFG-POp	NA
	Sham	40	27/13	59.80 (5.91)	10.38 (2.76) (d)						
	Placebo	40	24/16	59.73 (5.82)	10.37 (2.8) (d)						
Hai-mei L 2019	H-TMS	40	23/17	63.25 (3.81)	6.98 (1.44) (d)	20 min	10 (2 w)	WAB	Chinese	IFG-POp	NA
	L-TMS	40	22/18	62.31 (3.64)	6.25 (1.31) (d)						
Zhao RX 2019	L-TMS	46	26/20	49.88 (7.19)	3.84 (1.02) (w)	10 min	2 m	WAB	Chinese	IFG-POp	NA
	H-TMS	46	25/21	50.14 (7.21)	3.91 (1.15) (w)						
Jiang XC 2023	iTBS	22	9/13	58.86 (12.81)	39.95 (28.84) (d)	200 s	24 (4 w)	CRRCAE	Chinese	IFG-POp	AL
	Sham	23	16/7	63.57 (9.69)	35.04 (30.65) (d)						
Chen Y 2020	L-TMS	14	9/5	56.6 (15.1)	9.5 (3.3) (w)	20 min	10 (2 w)	CRRCAE	Chinese	IFG-POp	AL
	Sham	15	12/3	61.9 (10.7)	10.7 (4.4) (w)						
Yang YJ 2021	L-TMS	20	14/6	52.45 (13.95)	8.98 (1.76) (m)	NM	10 (2 w)	WAB	Chinese	IFG-PTr	AL
	Sham	20	15/5	51.72 (14.25)	9.30 (2.64) (m)						
Yingna F 2016	L-TMS	58	32/26	64.4 (14.5)	6.9 (3.3) (d)	20 min	30 d	WAB	Chinese	IFG-POp	AL
	Placebo	58	33/25	65.4 (15.9)	7.2 (3.1) (d)						
Hu RL 2020	L-TMS	58	32/26	59.7 (3.2)	2.6 (1.2) (m)	NM	15 (1 m)	ABC	Chinese	NA	NA
	Placebo	58	30/28	59.6 (3.3)	2.7 (1.1) (m)						
Wang JR 2018	L-TMS	15	11/4	47.07 (12.52)	39.40 (24.05) (d)	20 min	20 (4 w)	ABC	Chinese	IFG-POp	AL
	Sham	15	10/5	55.00 (11.35)	40.87 (21.86) (d)						
Du Y 2023	H-TMS & L-TMS	15	12/3	67.67 (13.69)	15.60 (1.64) (d)	20 min	10 (2 w)	ABC	Chinese	IFG	NA
	L-TMS	15	11/4	63.73 (11.76)	15.53 (1.88) (d)						
	Sham	15	8/7	64.27 (11.51)	15.13 (1.64) (d)						
Seniów J 2013	L-TMS	20	8/12	61.8 (11.8)	33.5 (24.1) (d)	30 min	15 (3 w)	BDAE	Polish	IFG-PTr	AL
	Sham	20	10/10	59.7 (0.7)	39.9 (28.9) (d)						
Waldowski K 2012	L-TMS	13	6/7	62.31 (11.03)	28.92 (19.39) (d)	30 min	15 (3 w)	BDAE	Polish	IFG-PTr & POp	AL
	Sham	13	7/6	60.15 (10.58)	48.54 (32.33) (d)						

AAT, Aachener Aphasie Test; ABC, Aphasia Battery of Chinese; BDAE, Boston Diagnostic Aphasia Examination; CCAT, Concise Chinese Aphasia Test; CRRCAE, Chinese Rehabilitation Research Center aphasia examination; WAB, Western Aphasia Battery; CRRCAE, Chinese Rehabilitation Research Center Aphasia Examination; d, day; w, week; m, month; NM, not mentioned; IFG, inferior frontal gyrus; SFG, superior frontal gyrus; STG, superior temporal gyrus; POp, pars opercularis; PTr, pars triangularis; AL, anatomical landmarks; NN, neuronavigational system.

Regarding therapeutic interventions, LF-rTMS was the most frequently employed approach. Treatment session duration typically ranged from 10 to 30 min. Notably, cTBS and iTBS sessions were shorter, lasting approximately 3 min. The overall treatment duration across studies ranged from 10 to 30 days. The commonly used assessment scale is WAB, of which there are 23 studies with the outcome indicator of WAB, 5 studies (36, 39, 42, 47, 55) with the outcome indicator is ABC, 4 literature’s outcome indicator is CCAT (26, 28, 29, 32), 2 literature (33, 45) used CRRCAE to assess the treatment effect, 2 literature (20, 27) used BDAE to evaluate the efficacy, and 1 literature (19) used AAT to assess. Most of the literature was collected from patients with Chinese as their first native language and also covered languages such as German, Farsi, and Polish.

### 3.3 Risk of bias assessment

**Figure 2:**
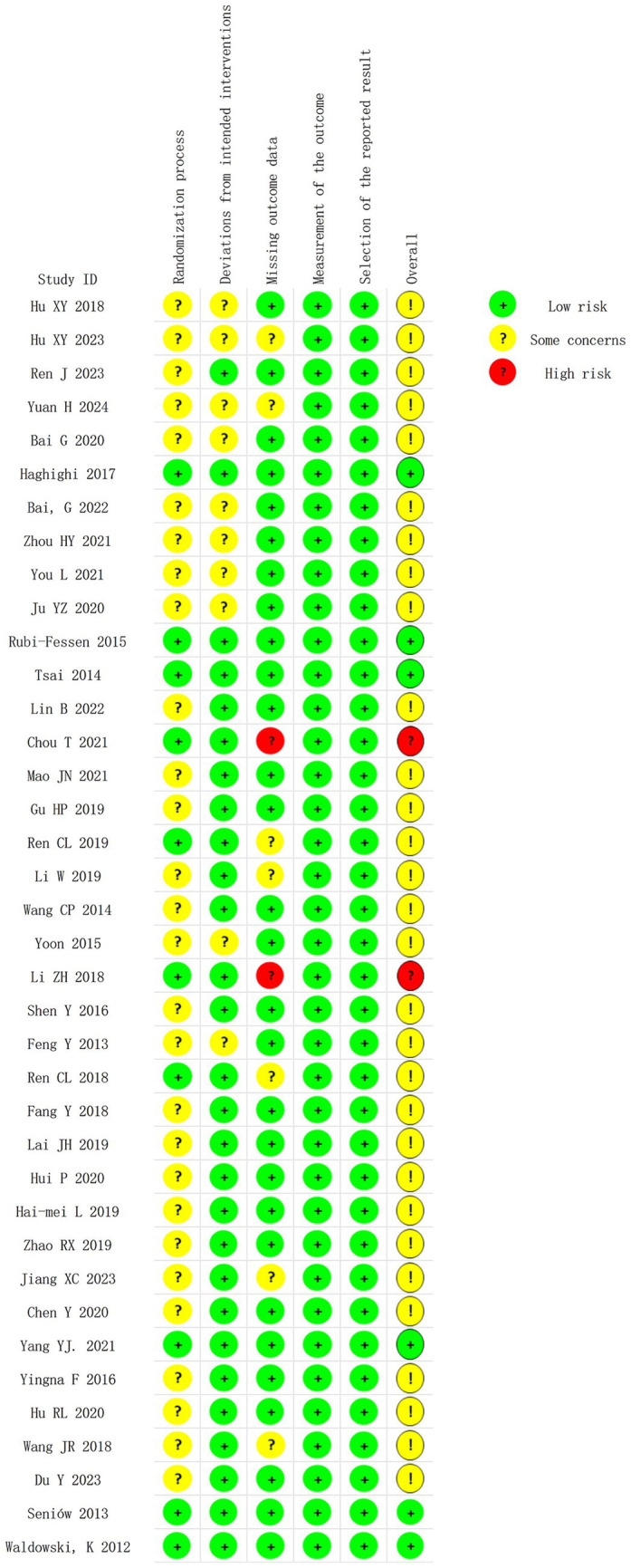
Risk of bias summary of included studies.

**Figure 3:**
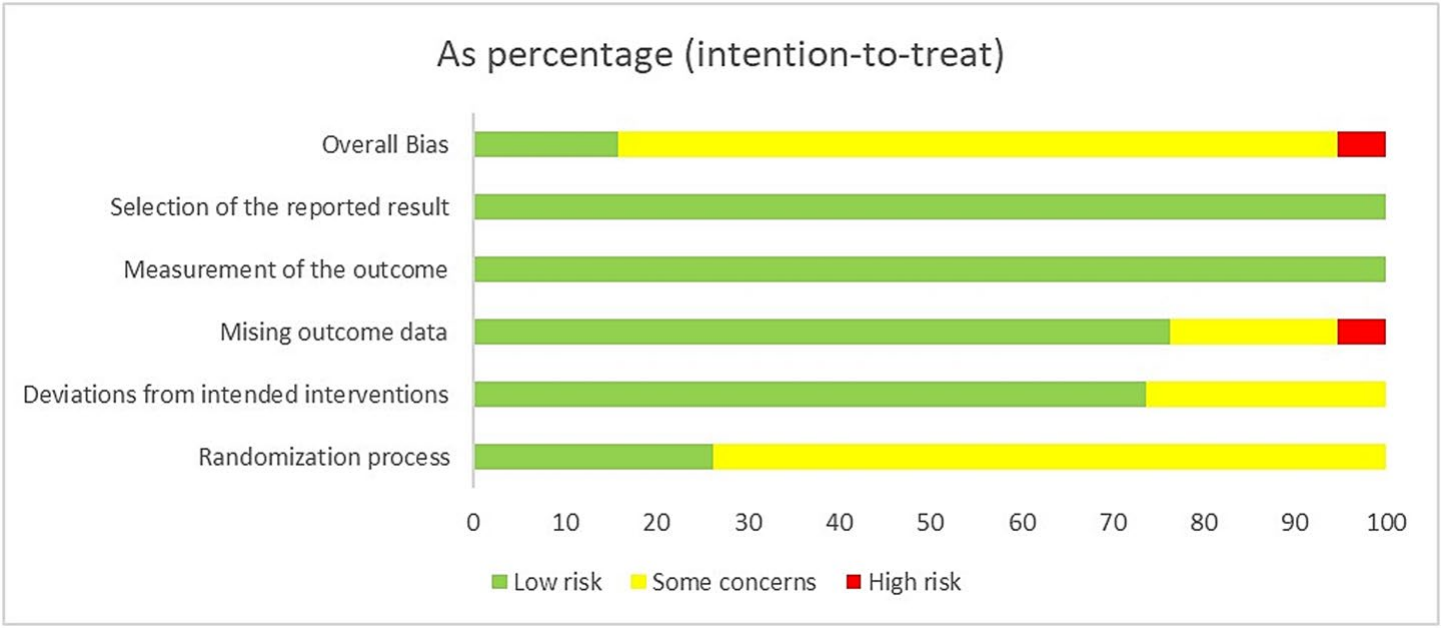
Proportion of risk levels of bias in each domain.

Figures 2, 3 summarize the risk of bias for the 38 RCTs included in this study. Two trials were judged as having a high risk of bias due to missing data, and there is no evidence that the results were not affected by the missing data. Thirty trials raised some concerns due to blinding issues or missing data issues, and 6 trials (19, 20, 26, 27, 44) were assessed as having a low risk of bias.

### 3.4 Results of NMA

#### 3.4.1 Primary outcome

##### 3.4.1.1 Network structure and geometry

**Figure 4:**
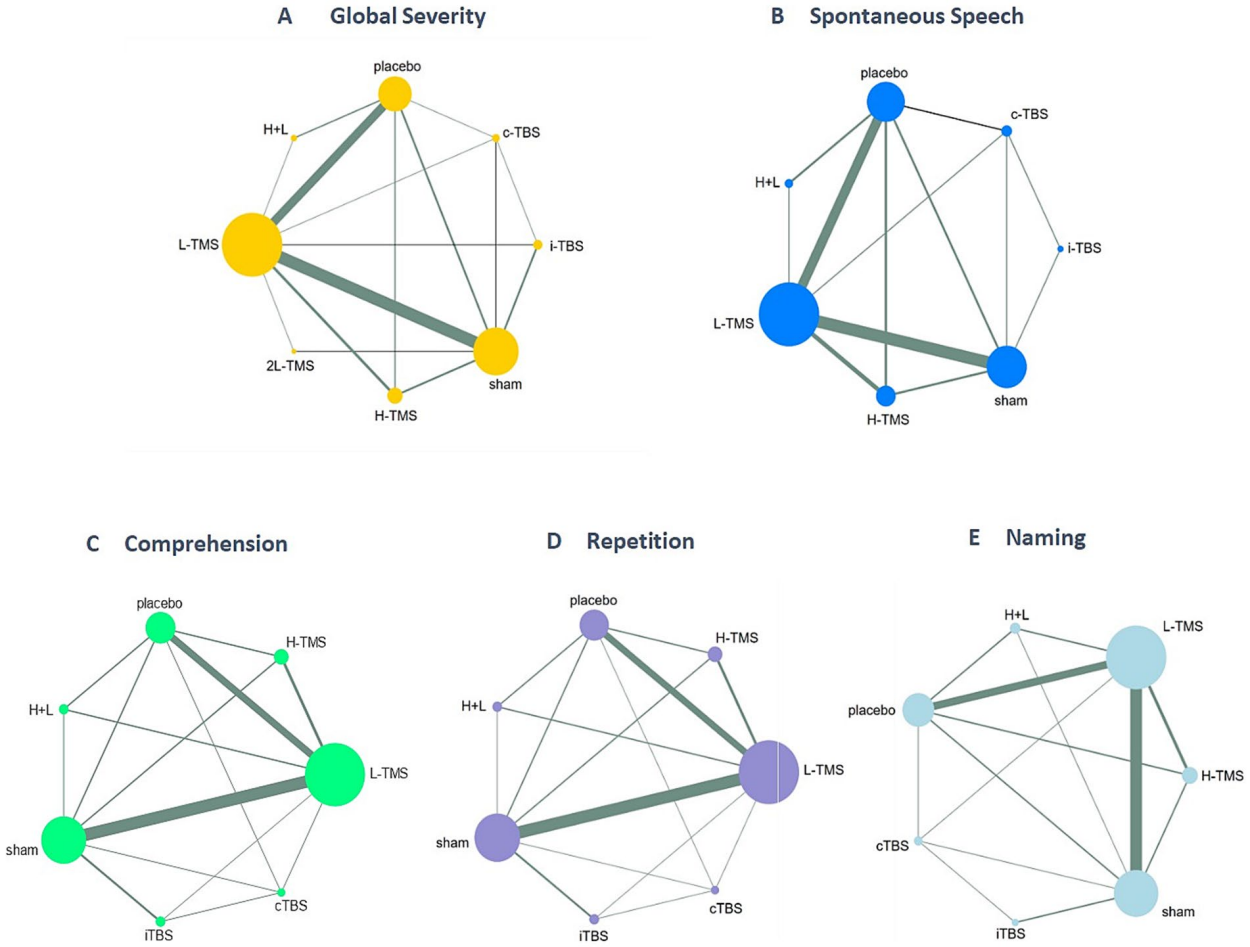
Network geometry of interventions across each language domain. TMS, repetitive transcranial magnetic stimulation; TBS, theta burst stimulation; L-TMS, low-frequency TMS; H-TMS, high-frequency TMS; 2L-TMS, twice-daily low-frequency TMS; H + L, L-TMS in combination with H-TMS; iTBS, intermittent theta burst stimulation; cTBS, continuous theta burst stimulation; sham, sham stimulation; placebo, control group. **A**: global severity; **B**: spontaneous speech; **C**: comprehension; **D**: repetition; **E**: naming.

Figure 4 shows network diagrams detailing comparisons of interventions for global severity of aphasia (Figure 4A), spontaneous speech (Figure 4B), comprehension (Figure 4C), repetition (Figure 4D), and naming (Figure 4E). Trials for global aphasia severity contained 8 nodes with 17 direct comparisons; spontaneous speech contained 7 nodes with 13 direct comparisons; comprehension contained 7 nodes with 15 direct comparisons; repetition contained 7 nodes with 15 direct comparisons; and naming contained 7 nodes with 14 direct comparisons. Regarding node size, LF-rTMS represented the most extensively investigated intervention, while all networks contained closed loops.

##### 3.4.1.2 Inconsistency checks

The results of the global inconsistency tests showed no inconsistency between the included studies for global severity of aphasia (*p* = 0.6126), spontaneous speech (*p* = 0.8609), comprehension (*p* = 0.8563), repetition (*p* = 0.6832), and naming (*p* = 0.5462), suggesting that a consistency models was chosen for data analysis. Local inconsistency, evaluated via the node-splitting approach, yielded non-significant *p*-values (*p* > 0.05) for all comparisons, indicating agreement between direct and indirect evidence. Loop-specific inconsistency was further quantified using the inconsistency factor (IF). The IF values across all closed loops ranged from 0.00 to 1.32. All 95% confidence intervals for these IF estimates included 0 (indicating no inconsistency), except for one loop in the confrontation naming network (involving interventions B-F-G; lower 95% CI limit: 0.11). None of the loop-specific tests reached statistical significance (*p* > 0.05), confirming the absence of significant local inconsistency.

##### 3.4.1.3 Network estimates

**Figure 5:**
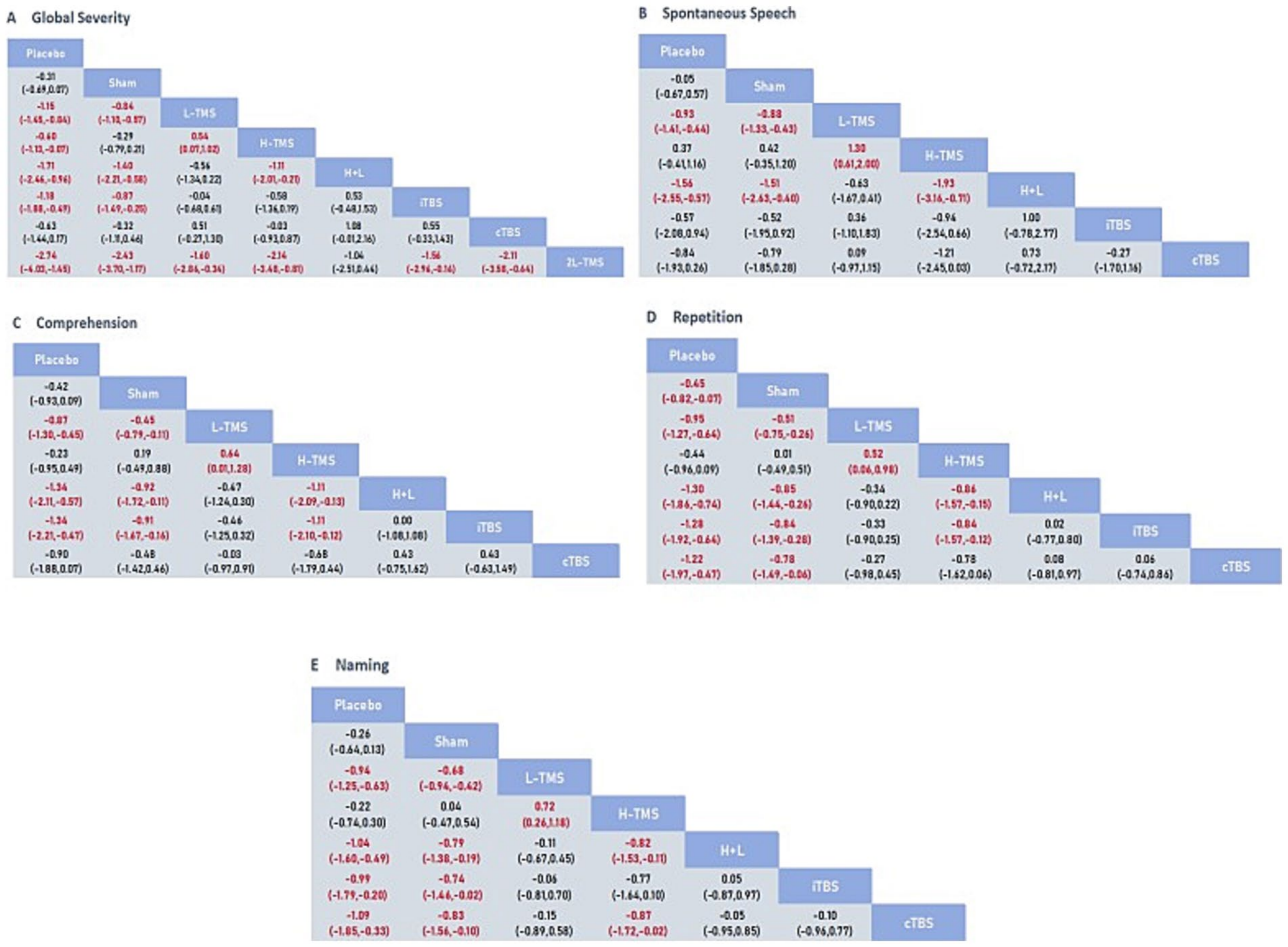
League table for all mean differences and 95% credible interval (CrI). **A**: global severity; **B**: spontaneous speech; **C**: comprehension; **D**: repetition; **E**: naming.

**Figure 6:**
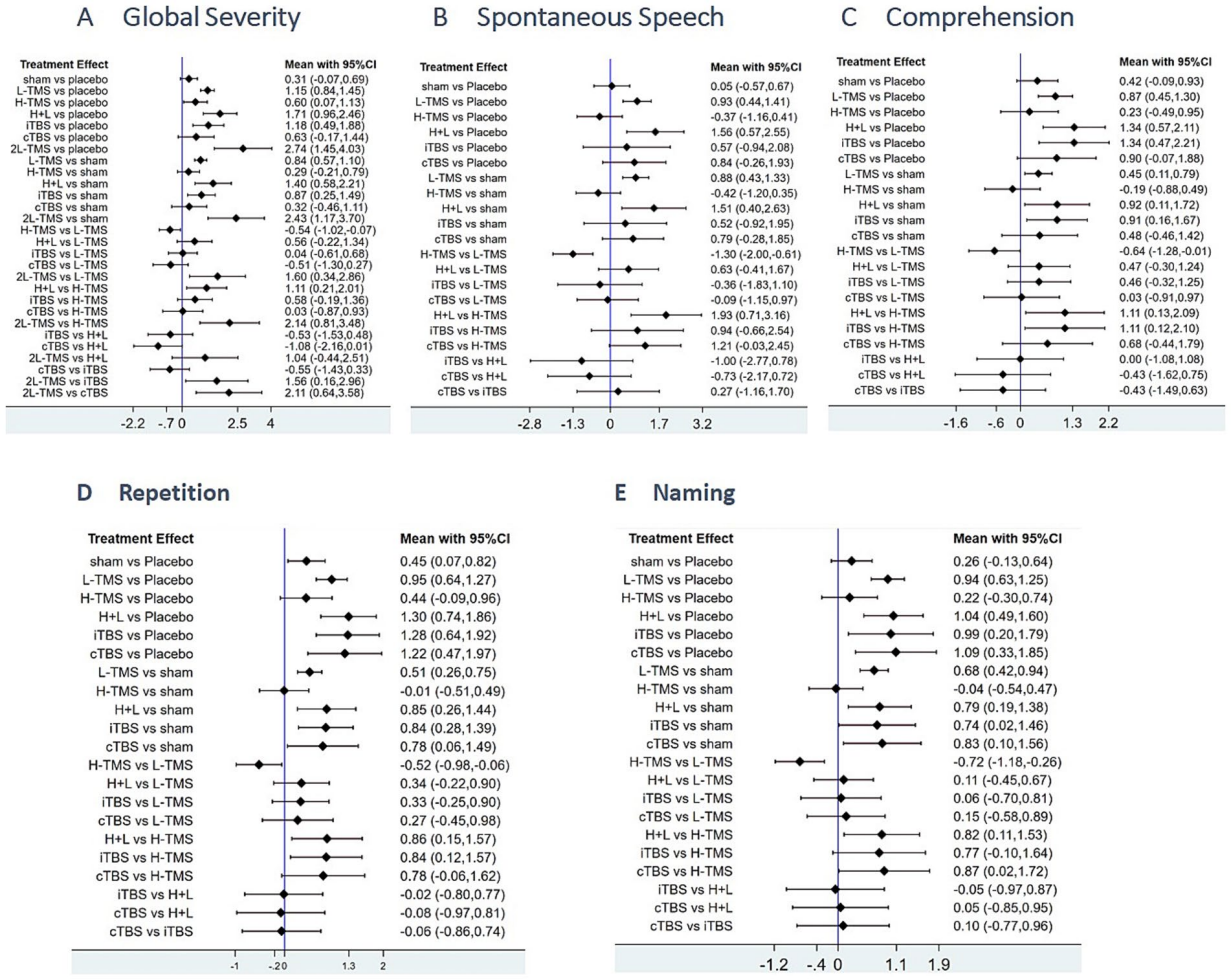
Forest plots of the posterior mean difference and 95% credible intervals. **A**: global severity; **B**: spontaneous speech; **C**: comprehension; **D**: repetition; **E**: naming.

Figure 5 presents the posterior mean differences with 95% confidence intervals for all pairwise comparisons, where statistically significant results are highlighted in red. Figure 6 displays the forest plots for each outcome measure, showing effect estimates with corresponding 95% confidence intervals.

###### 3.4.1.3.1 Global severity

A total of 34 studies were published on the global severity of aphasia, and 8 interventions were included. For the global severity of aphasia, compared with the control group or the sham stimulation group, LF-rTMS, LF-rTMS & HF-rTMS, iTBS, and 2LF-rTMS were able to improve the global severity of patients with aphasia after stroke. 2LF-rTMS was more effective in improving the global severity of patients with aphasia after stroke, compared with LF-rTMS, LF-rTMS & HF-rTMS, and iTBS (see Figures 5A, 6A).

###### 3.4.1.3.2 Spontaneous speech

Twenty-two studies assessing spontaneous speech outcomes evaluated seven interventions. In terms of spontaneous speech, LF-rTMS and LF-rTMS & HF-rTMS were significantly more effective when comparing the control and sham stimulation groups. There was no statistical significance in the comparisons between the control groups and sham stimulation groups (see Figures 5B, 6B).

###### 3.4.1.3.3 Comprehension

Thirty-three studies evaluating auditory comprehension outcomes assessed seven interventions. Compared to control/sham stimulation, LF-rTMS, LF-rTMS & HF-rTMS, and iTBS demonstrated significant improvements. However, pairwise comparisons between these three active interventions showed no statistically significant differences (see Figures 5C, 6C).

###### 3.4.1.3.4 Repetition

Thirty-one studies assessing repetition outcomes evaluated seven interventions. Significant improvements versus control/sham groups were observed for LF-rTMS, LF-rTMS & HF-rTMS, iTBS, and cTBS. However, no statistically significant differences emerged in direct pairwise comparisons between these four active interventions. At the same time, the effect of the sham stimulation group on repetition was also statistically significant compared with the control group (see Figures 5D, 6D).

###### 3.4.1.3.5 Naming

Twenty-nine studies evaluating confrontation naming outcomes assessed seven interventions. Compared to control/sham stimulation, LF-rTMS, HF-rTMS & LF-rTMS, iTBS, and cTBS demonstrated statistically significant improvements. However, no significant differences were observed in pairwise comparisons between these active interventions (see Figures 5E, 6E).

##### 3.4.1.4 Ranking probabilities

**Figure 7:**
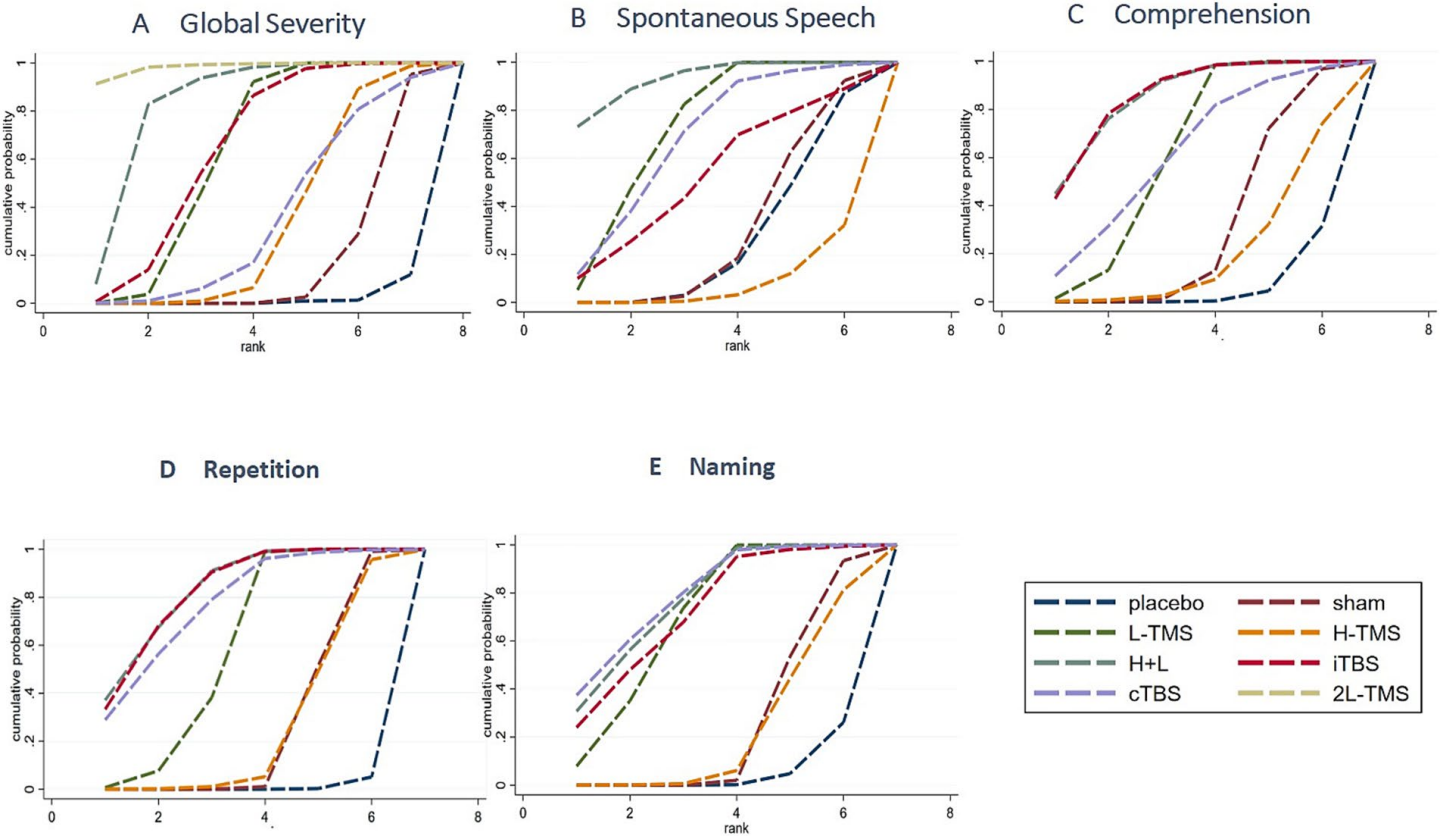
Probability of ranking for each intervention for outcomes. **A**: global severity; **B**: spontaneous speech; **C**: comprehension; **D**: repetition; **E**: naming.

Based on SUCRA (surface under the cumulative ranking curve) values, 2LF-rTMS was most likely to be ranked first for relieving global aphasia severity, followed by LF-rTMS & HF-rTMS (see Figure 7A). Regarding spontaneous speech improvement, LF-rTMS & HF-rTMS demonstrated the highest probability of ranking first, with LF-rTMS ranking second (see Figure 7B). For comprehension, LF-rTMS & HF-rTMS and iTBS were most likely to rank first, followed by LF-rTMS and cTBS (see Figure 7C). For repetition, LF-rTMS & HF-rTMS and iTBS were the most effective interventions, followed by cTBS (see Figure 7D). For naming, cTBS was the most effective interventions, followed by LF-rTMS & HF-rTMS (see Figure 7E).

##### 3.4.1.5 Sensitivity analysis

###### 3.4.1.5.1 Global severity

Due to the limited number of studies investigating specific interventions—namely, only one study (21) evaluating dual-LF-rTMS (which yielded an exceptionally high SUCRA value) and only two studies evaluating cTBS—a sensitivity analysis was performed. After excluding these three studies, inconsistency was re-assessed. The results indicated no significant inconsistency (*p* = 0.5757), justifying the continued use of the consistency model. Under this model, combined LF- and HF-rTMS remained the intervention most likely to rank first for the primary outcome, consistent with the original findings. This suggests that the overall results of this network meta-analysis are robust.

###### 3.4.1.5.2 Spontaneous speech

Given the limited evidence for specific interventions—notably, only two studies investigating LF-rTMS & HF-rTMS (yielding high SUCRA values) and fewer than three studies each for iTBS and cTBS—a sensitivity analysis was conducted by excluding these five studies. Following exclusion, inconsistency assessment of the remaining network revealed no significant inconsistency (*p* = 0.4155). Consequently, the consistency model was retained. Under this model, LF-rTMS remained the intervention most likely to rank first for the primary outcome, consistent with the original findings. This indicates that the core results of this network meta-analysis are robust.

###### 3.4.1.5.3 Comprehension

Due to the limited number of studies evaluating cTBS (only two studies, both yielding high SUCRA values), a sensitivity analysis was conducted by excluding these two studies. Subsequent inconsistency assessment of the remaining network indicated no significant inconsistency (*p* = 0.7790). Therefore, the consistency model was retained. Under this model, LF-rTMS & HF-rTMS and iTBS remained the interventions with the highest probability of being the most efficacious for the primary outcome, consistent with the original findings. This indicates that the key results of this network meta-analysis are robust.

###### 3.4.1.5.4 Repetition

After excluding the two studies investigating cTBS, the assessment of inconsistency between direct and indirect evidence indicated no significant inconsistency (*p* = 0.5600). Consequently, the consistency model was retained for data analysis. Within this model, both LF-rTMS & HF-rTMS and iTBS remained the interventions demonstrating the highest efficacy for the primary outcome, consistent with the original findings. This further supports the robustness of the key results in this network meta-analysis.

###### 3.4.1.5.5 Naming

After excluding the four studies investigating cTBS and iTBS, the assessment of inconsistency between direct and indirect evidence revealed no significant inconsistency (*p* = 0.6260). Consequently, the consistency model was applied for analysis. Under this model, LF-rTMS & HF-rTMS remained the intervention demonstrating the highest efficacy for the primary outcome, consistent with the original findings. This indicates enhanced robustness of the core results in this network meta-analysis.

##### 3.4.1.6 Publication bias

**Figure 8:**
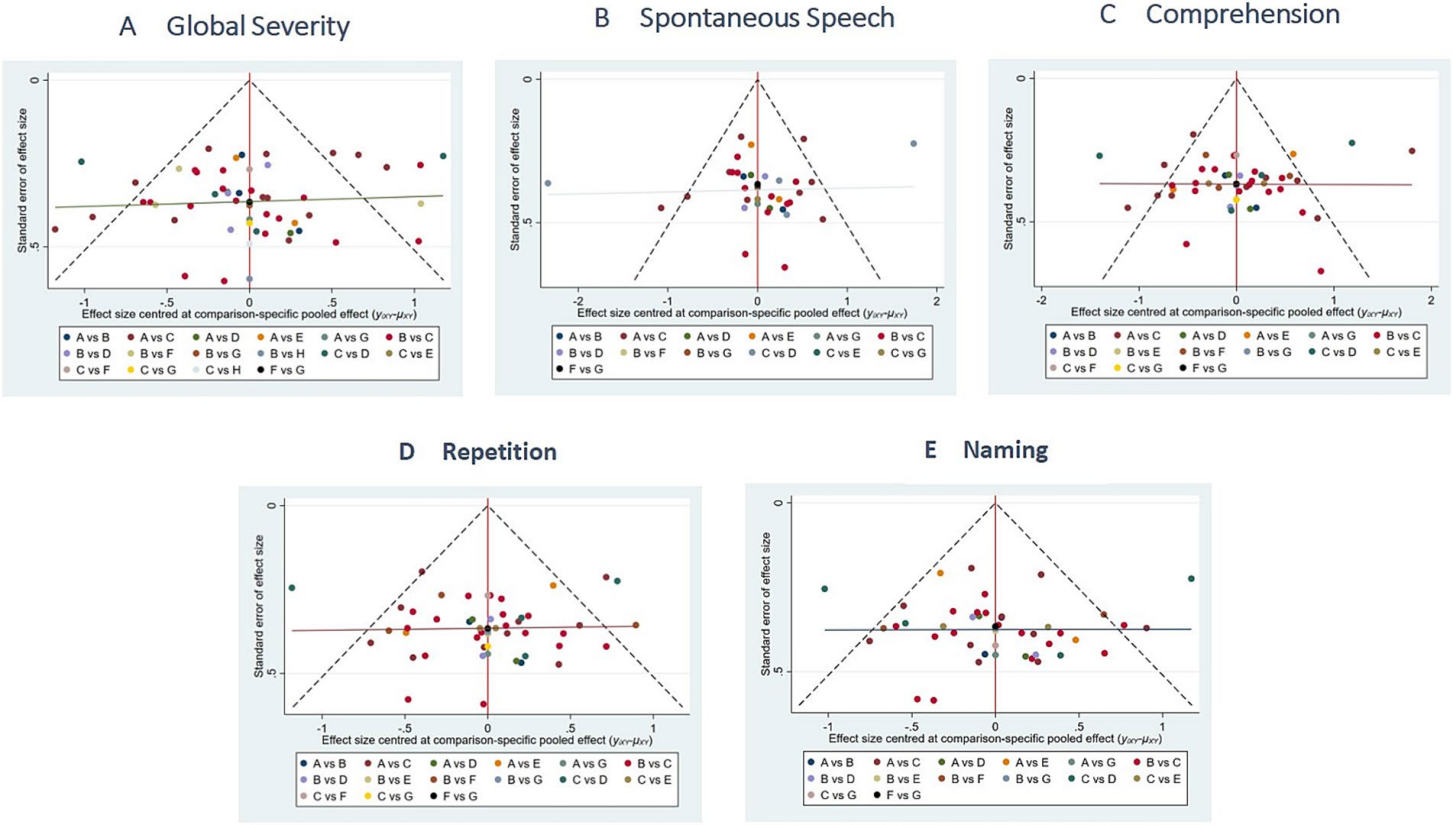
Comparison of outcome indicators—corrected inverted funnel plot. **A**: global severity; **B**: spontaneous speech; **C**: comprehension; **D**: repetition; **E**: naming.

The funnel plots exhibited substantial symmetry, indicating a balanced distribution of included studies. However, the presence of a few data points near the lower and outer margins of the funnel may indicate potential publication bias or small-study effects (see Figure 8).

#### 3.4.2 Secondary outcome

##### 3.4.2.1 Stimulation site

Following the exclusion of studies that did not specify the precise stimulation site or had fewer than three studies investigating a given site. A total of 30 studies were ultimately included in the analysis. The stimulation sites targeted the PTr and the POp of IFG.

###### 3.4.2.1.1 Network structure and geometry

**Figure 9:**
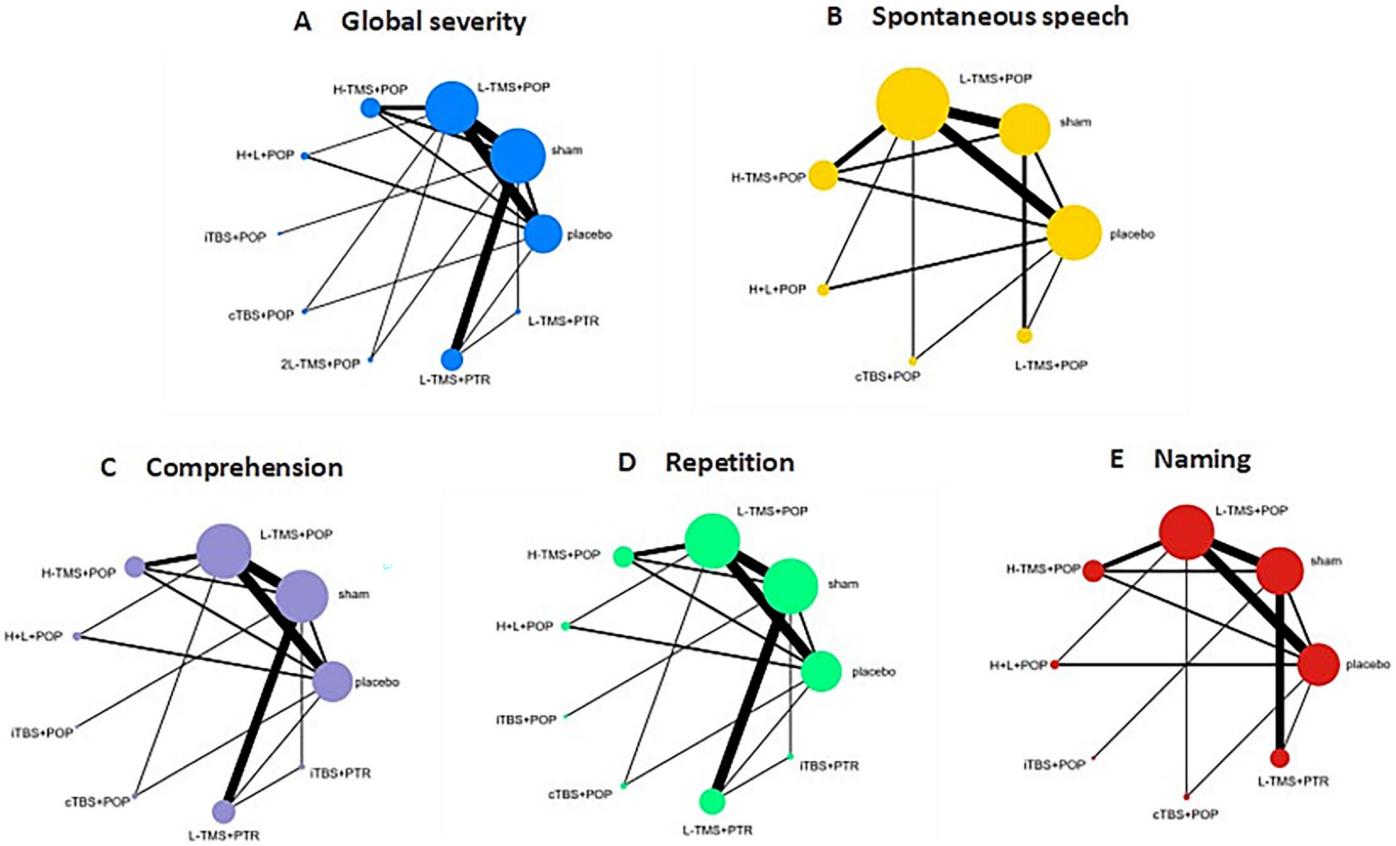
Network geometry (stimulation site). TMS, repetitive transcranial magnetic stimulation; TBS, theta burst stimulation; L-TMS, low-frequency TMS; H-TMS, high-frequency TMS; 2L-TMS, twice-daily low-frequency TMS; H + L, L-TMS in combination with H-TMS; iTBS, intermittent theta burst stimulation; cTBS, continuous theta burst stimulation; sham, sham stimulation; placebo, control group; POp, pars opercularis; PTR, pars triangularis. **A**: global severity; **B**: spontaneous speech; **C**: comprehension; **D**: repetition; **E**: naming.

Figure 9 presents network diagrams illustrating the comparisons of interventions. For global severity of aphasia (Figure 9A), spontaneous speech (Figure 9B), comprehension (Figure 9C), repetition (Figure 9D), and naming (Figure 9E). Trials for global Severity of aphasia contained 10 nodes with 16 direct comparisons; spontaneous speech contained 7 nodes with 12 direct comparisons; comprehension contained 9 nodes with 15 direct comparisons; repetition contained 9 nodes with 15 direct comparisons; and naming contained 8 nodes with 13 pairs of comparisons. Regarding node size, the intervention of low-frequency stimulation applied to the POp of IFG corresponded to the largest node, indicating it was the most represented intervention across the included studies.

###### 3.4.2.1.2 Network estimates

**Figure 10:**
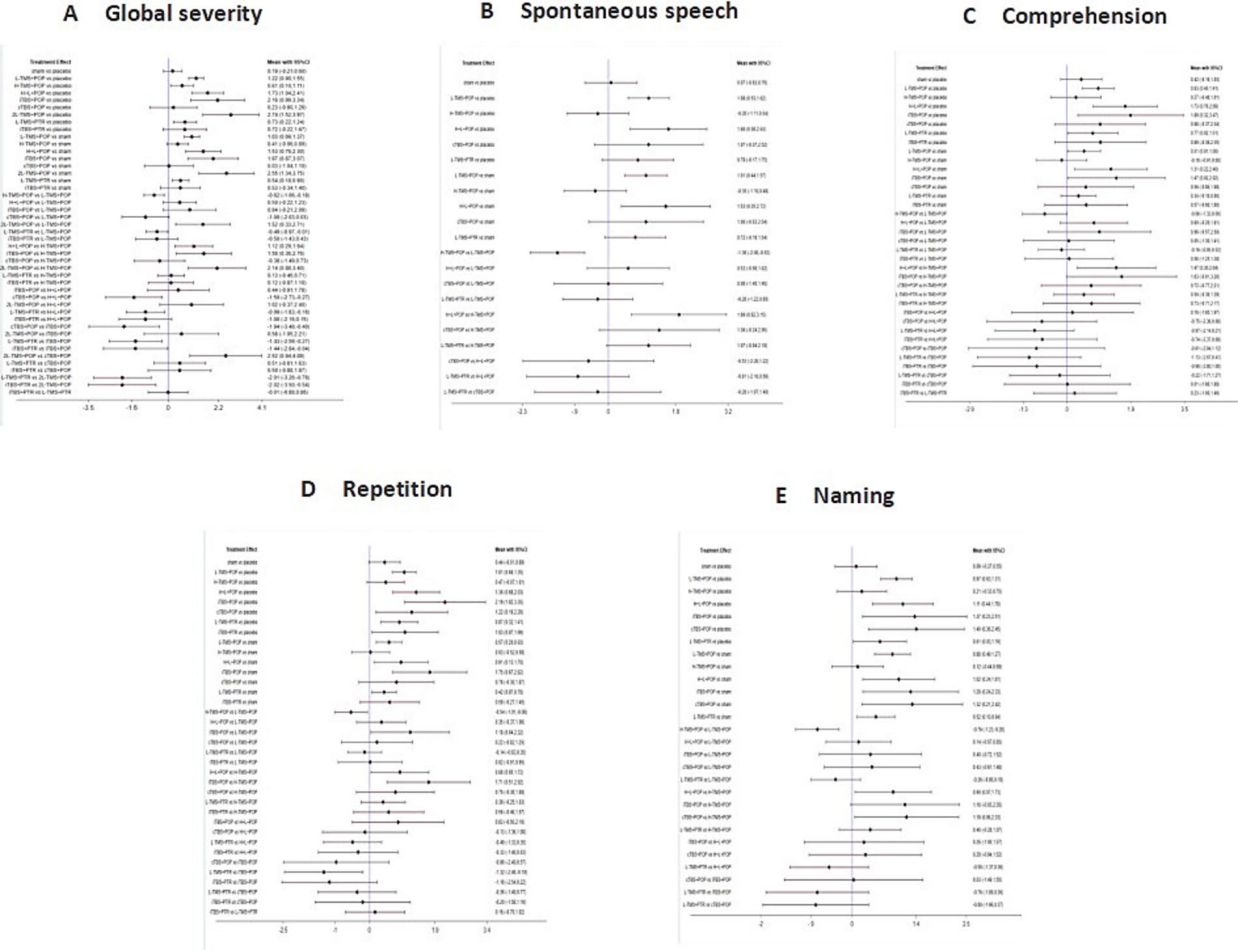
Forest plots of the posterior mean difference and 95% credible intervals (stimulation site). **A**: global severity; **B**: spontaneous speech; **C**: comprehension; **D**: repetition; **E**: naming.

Forest plot analysis revealed statistically significant differences for specific comparisons: LF-rTMS applied to POp (LF-TMS + POp) vs. LF-rTMS applied to PTr (LF-TMS + PTr) was statistically significant, while not statistically significant in the other four dimensions. iTBS applied to POp (iTBS + POp) versus iTBS applied to PTr (iTBS + PTr) in the global severity of aphasia was also statistically significant, but did not show statistical significance in comprehension and repetition (see Figure 10).

###### 3.4.2.1.3 Ranking probabilities

**Figure 11:**
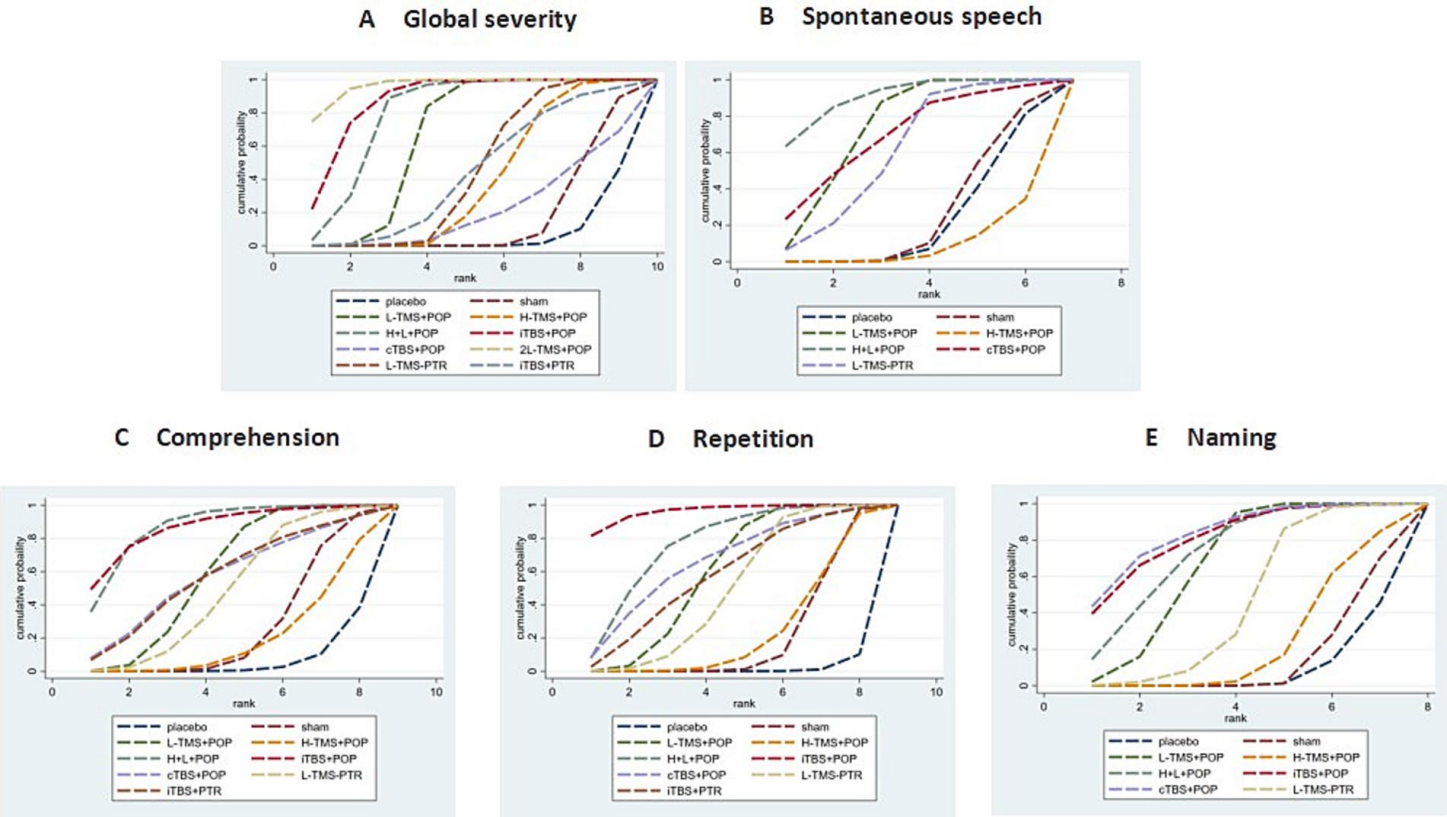
Probability of ranking for each intervention for outcomes (stimulation site). **A**: global severity; **B**: spontaneous speech; **C**: comprehension; **D**: repetition; **E**: naming.

Based on the SUCRA values, 2LF-rTMS + POp demonstrated the highest probability of ranking first in alleviating the global severity of aphasia, followed by iTBS + POp (Figure 11A). For spontaneous speech, most likely to rank first was LF-rTMS & HF-rTMS + POp (Figure 11B). For comprehension, LF-rTMS & HF-rTMS + POp and iTBS + POp were most likely to rank first (Figure 11C). For repetition, LF-rTMS & HF-rTMS + POp and iTBS + POp were the most effective (Figure 11D); for naming, cTBS + POp was the most effective, followed by iTBS + POp (Figure 11E). Notably, across the individual functional metrics, LF-TMS + POp consistently demonstrated superior therapeutic efficacy compared to both LF-TMS + PTr.

###### 3.4.2.1.4 Sensitivity analysis

Sensitivity analyses were performed by excluding studies with fewer than three interventions and studies containing interventions associated with abnormally high SUCRA values. Re-running the analyses yielded results consistent with the primary findings, suggesting that the overall results of this study are robust.

###### 3.4.2.1.5 Publication bias

**Figure 12:**
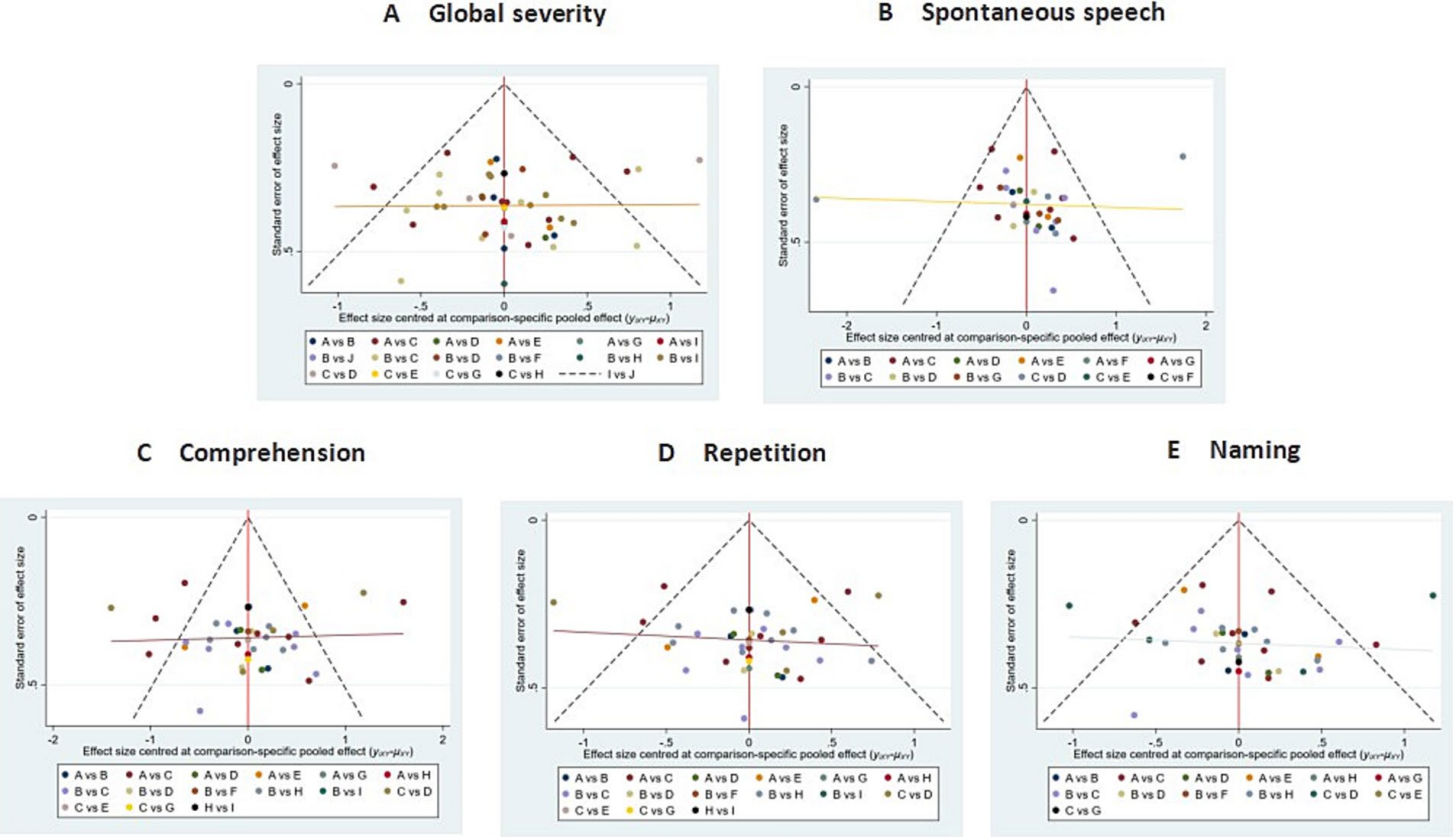
Comparison of outcome indicators—corrected inverted funnel plot (stimulation site). **A**: global severity; **B**: spontaneous speech; **C**: comprehension; **D**: repetition; **E**: naming.

Funnel plot symmetry was generally observed, indicating a balanced distribution of included studies. The presence of data points in the lower periphery, however, may indicate potential publication bias or small-study effects (see Figure 12).

##### 3.4.2.2 Targeting method

Studies that did not explicitly report the localization method were excluded. The 10–20 EEG system was categorized under the anatomical landmarks (AL) localization method. Following this, a total of 26 studies were included, which utilized two localization methods: anatomical landmarks (AL) and neuronavigational system (NN).

###### 3.4.2.2.1 Network structure and geometry

**Figure 13:**
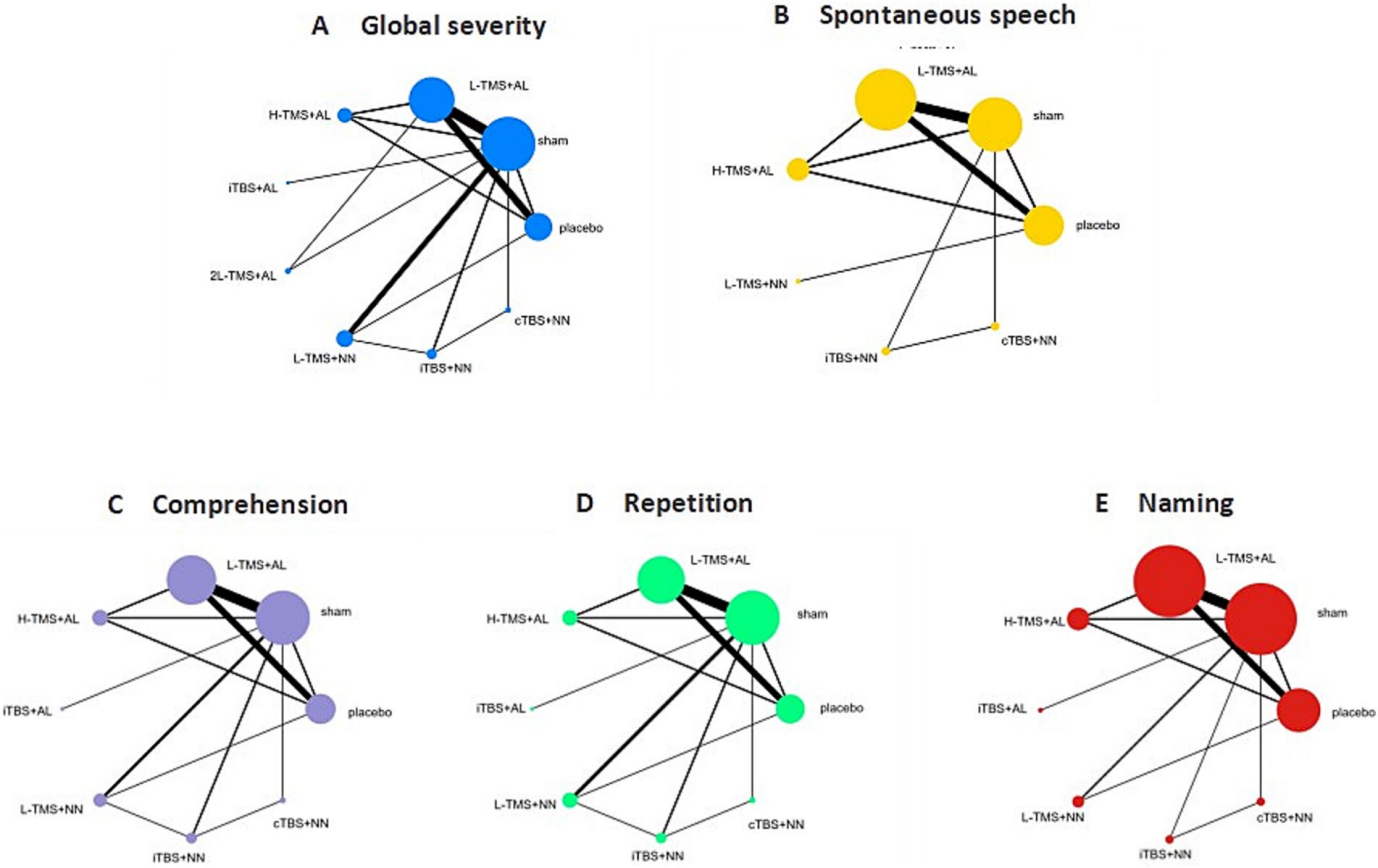
Network geometry (targeting method). TMS, repetitive transcranial magnetic stimulation; TBS, theta burst stimulation; L-TMS, low-frequency TMS; H-TMS, high-frequency TMS; 2L-TMS, twice-daily low-frequency TMS; H + L, L-TMS in combination with H-TMS; iTBS, intermittent theta burst stimulation; cTBS, continuous theta burst stimulation; sham, sham stimulation; placebo, control group; AL, location of anatomical landmarks; NN, neuronavigational system. **A**: global severity; **B**: spontaneous speech; **C**: comprehension; **D**: repetition; **E**: naming.

Figure 13 presents the network diagrams detailing the treatment comparisons for the global severity of aphasia (Figure 13A), spontaneous speech (Figure 13B), comprehension (Figure 13C), repetition (Figure 13D), and naming (Figure 13E). The corresponding networks comprised the following nodes and direct comparison pairs: global severity of aphasia (9 nodes, 15 pairs), spontaneous speech (7 nodes, 10 pairs), comprehension (8 nodes, 12 pairs), repetition (8 nodes, 13 pairs), and naming (8 nodes, 12 pairs).

###### 3.4.2.2.2 Network estimates

**Figure 14:**
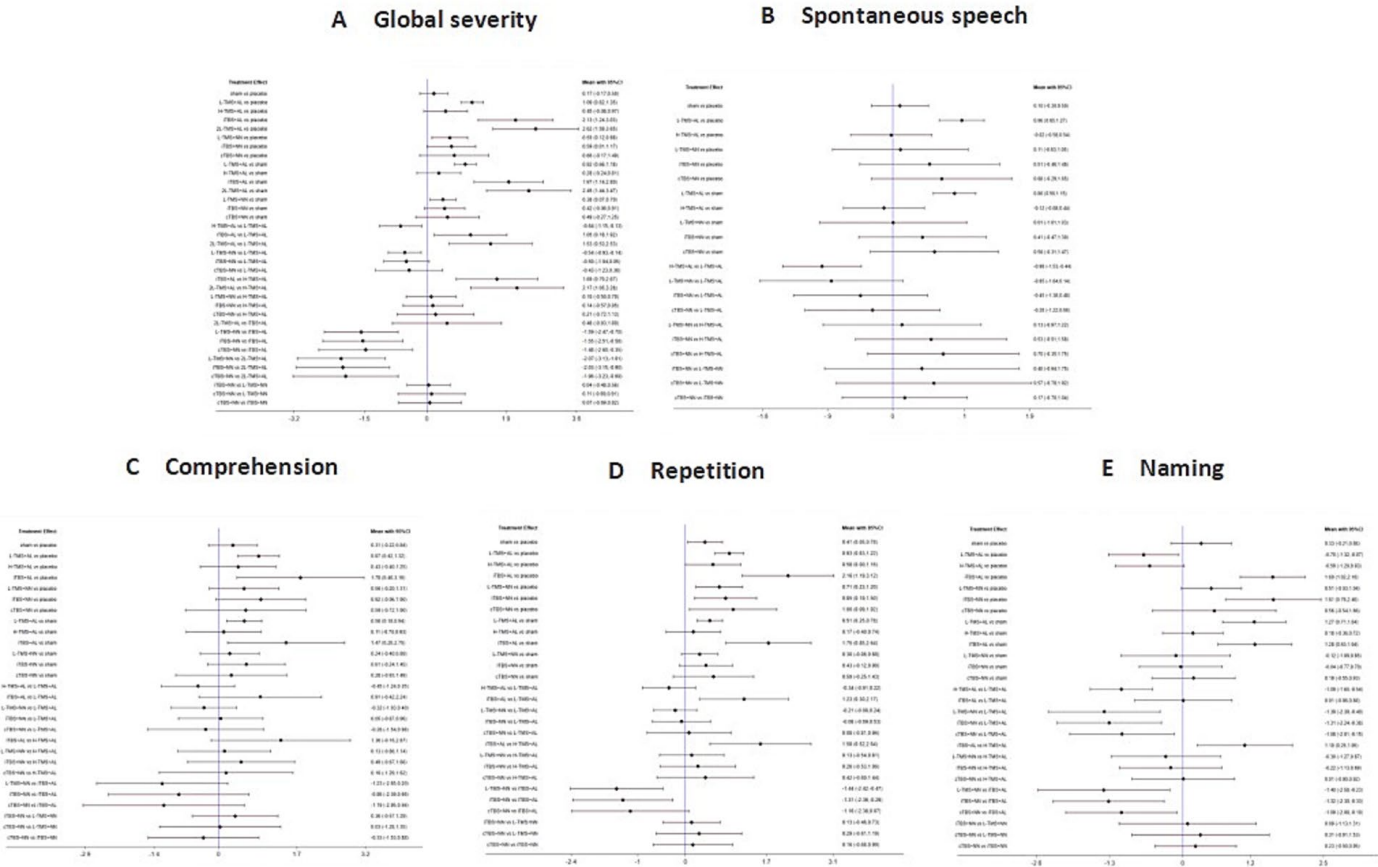
Forest plots of the posterior mean difference and 95% credible intervals (targeting method). **A**: global severity; **B**: spontaneous speech; **C**: comprehension; **D**: repetition; **E**: naming.

Forest plot analysis revealed the following statistically significant differences: LF-TMS + AL vs. LF-TMS + NN: significant in global severity of aphasia, but not significant in spontaneous speech, comprehension, or repetition. iTBS + AL vs. iTBS + NN: significant only in comprehension. No significant differences were observed for this comparison in global severity of aphasia, spontaneous speech, repetition, or naming (see Figure 14).

###### 3.4.2.2.3 Ranking probabilities

**Figure 15:**
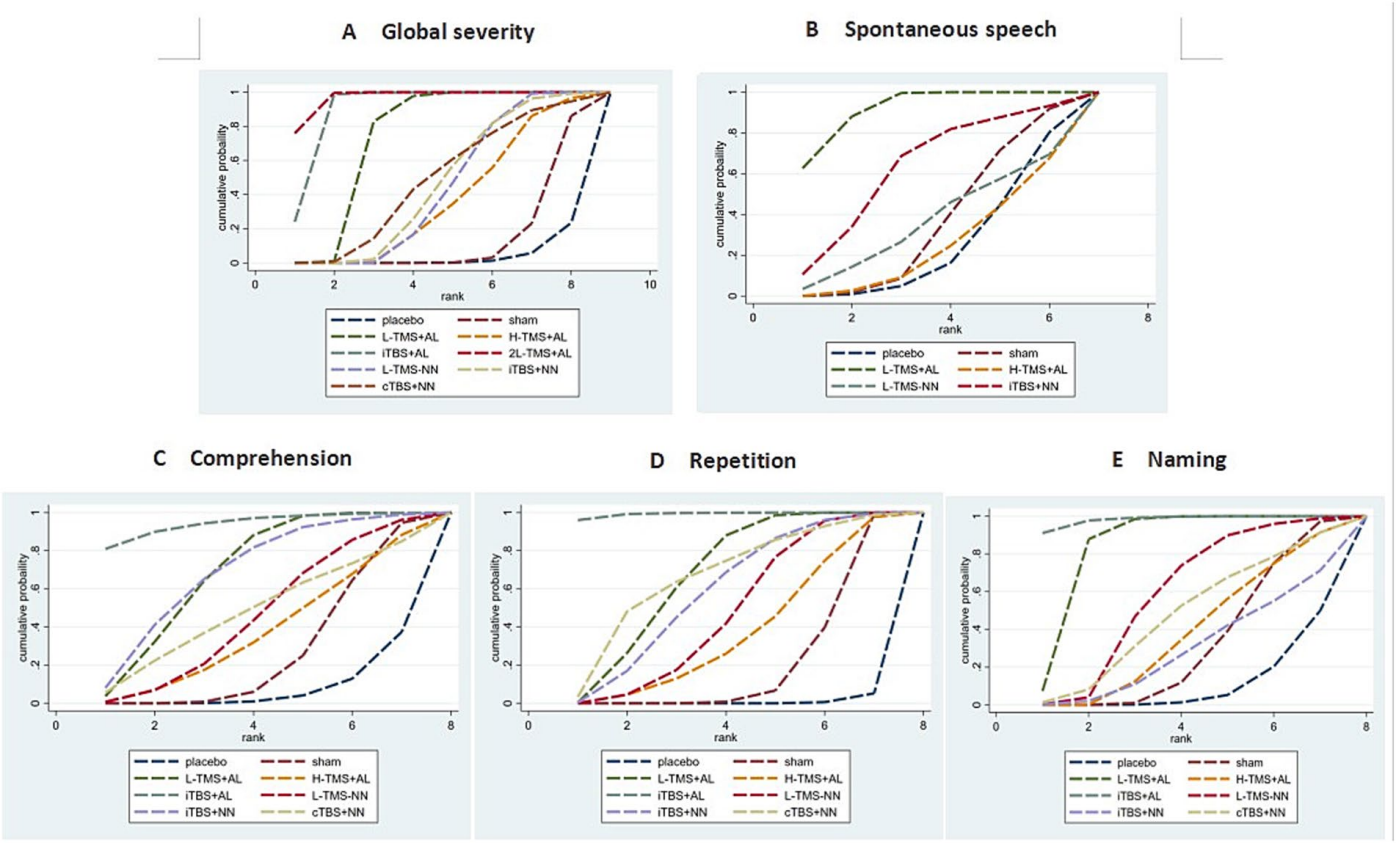
Probability of ranking for each intervention for outcomes (targeting method). **A**: global severity; **B**: spontaneous speech; **C**: comprehension; **D**: repetition; **E**: naming.

Based on the SUCRA values, LF-rTMS + AL demonstrated the highest probability of ranking first for alleviating global aphasia severity, followed by iTBS + AL (Figure 15A). Regarding spontaneous speech, LF-rTMS+AL was most likely to rank first (Figure 15B). For comprehension, iTBS + AL exhibited the highest likelihood of ranking first (Figure 15C). In repetition, iTBS + AL was the most effective intervention (Figure 15D), while for naming, iTBS + AL showed the most excellent efficacy, with LF-rTMS + AL ranking second (Figure 15E). Notably, across all functional domains, LF-rTMS + AL consistently demonstrated superior treatment efficacy relative to LF-rTMS + NN.

###### 3.4.2.2.4 Sensitivity analysis

Sensitivity analyses excluding studies with fewer than three interventions or containing interventions associated with abnormally high SUCRA values yielded results consistent with the primary findings, indicating the robustness of the study outcomes.

###### 3.4.2.2.5 Publication bias

**Figure 16:**
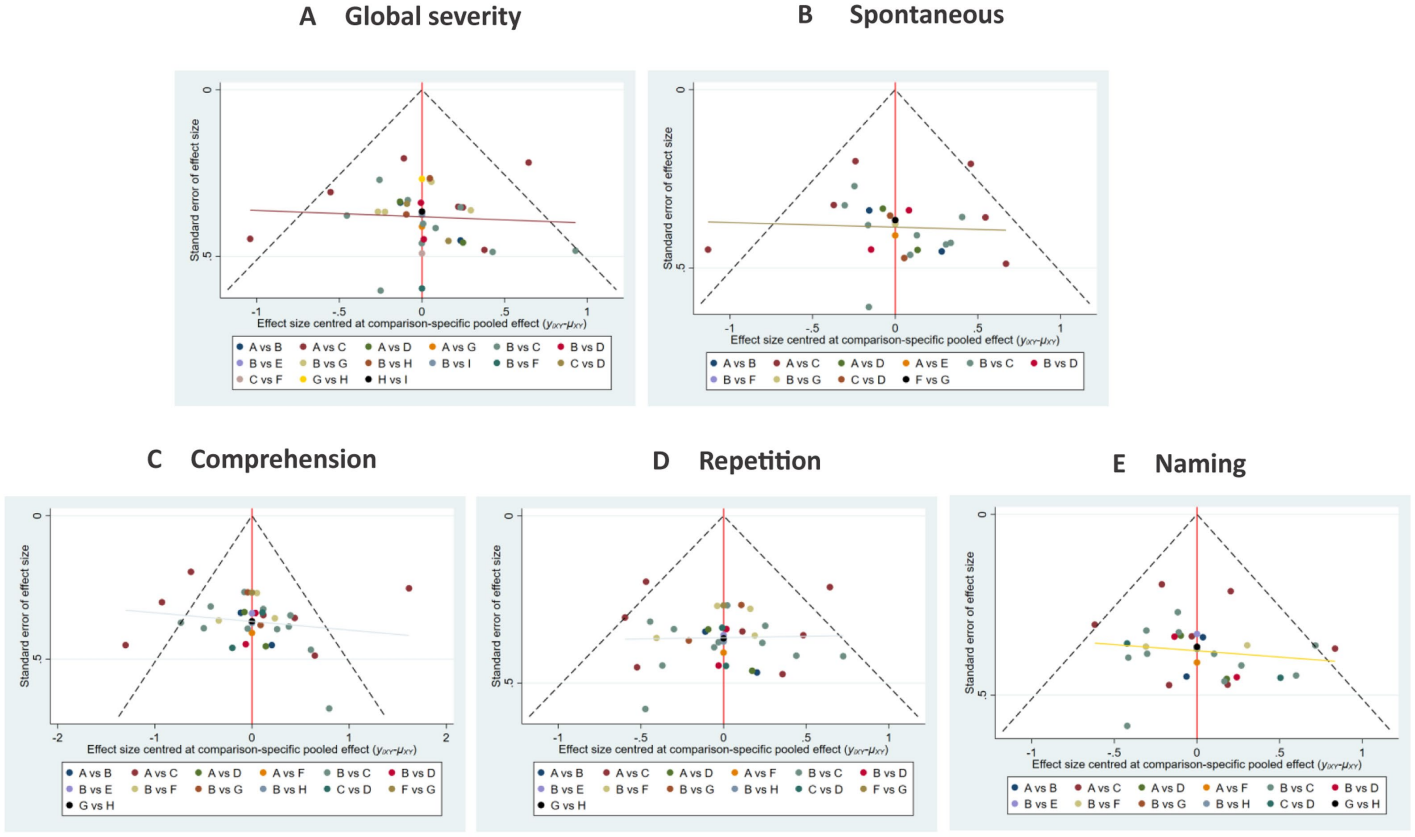
Comparison of outcome indicators—corrected inverted funnel plot (targeting method). **A**: global severity; **B**: spontaneous speech; **C**: comprehension; **D**: repetition; **E**: naming.

Funnel plots demonstrated broad symmetry, indicating a balanced distribution of included studies. However, several data points located in the lower periphery may suggest potential publication bias or small-study effects (see Figure 16).

#### 3.4.3 Adverse event

No serious adverse events were reported in the included studies. Among the 38 studies, 7 documented treatment-related adverse events. These predominantly occurred during transcranial magnetic stimulation sessions, manifesting as transient dizziness and headache. In one instance (24), the patient developed epilepsy unrelated to the treatment itself.

## 4 Discussion

This NMA represents the most exhaustive integration of rTMS, aiming to elucidate the effectiveness of different types of rTMS for post-stroke aphasia. The present study included 38 RCTs with 1982 participants, retrieved eight interventions, and systematically analyzed five core linguistic domains: global severity, spontaneous speech, comprehension, repetition, and naming. Consistency was observed between direct and indirect comparisons for all outcomes. Among the results analyzed, for alleviating the global severity of aphasia, 2LF-rTMS demonstrated optimal efficacy; for spontaneous speech, LF-rTMS & HF-rTMS showed superior efficacy; for comprehension, LF-rTMS & HF-rTMS and iTBS were the most effective; and for repetition, LF-rTMS & HF-rTMS and iTBS were the best. rTMS was superior to TBS in Global severity and spontaneous speech, and iTBS also showed better efficacy in comprehension and repetition, while cTBS was particularly effective in treating naming. LF-rTMS & HF-rTMS performed well in all aspects. The results of this study suggest that rTMS intervention is effective in the treatment of post-stroke aphasia and that different types of rTMS treatment show different advantages in different language domains. For the secondary outcome of stimulation site selection, a comparative analysis was conducted between the two subregions, POp and PTr. Based on superior efficacy, POp was selected. Regarding the localization method, AL and NN were compared, resulting in the determination that AL demonstrated superior efficacy.

The neural mechanisms underlying post-stroke language recovery are primarily conceptualized through two theoretical models: the compensatory model and the hemispheric competition model (57). The compensatory model posits that functional recovery involves the recruitment of neurons from regions adjacent to or remote from the lesion site to compensate for the damaged brain areas. The hemispheric competition model proposes that bilateral brain hemispheres maintain functional dynamic equilibrium through bidirectional inhibitory interactions mediated by the corpus callosum under normal physiological conditions. Following damage to the language-dominant (typically left) hemisphere, the functional capacity of the lesioned hemisphere diminishes. Concurrently, increased inhibitory drive from the contralateral (right) hemisphere further suppresses the excitability of the damaged hemisphere, resulting in pathological inhibition (13). The dominance of the post-stroke brain recovery model is closely related to the structural preservation of the brain, with the hemispheric competition model dominating in those with high structural conservation and the compensatory model dominating in those with low structural conservation; this is a “bidirectional equilibrium” recovery model.

Our findings align with recent studies (31, 48, 53) demonstrating the efficacy of LF-rTMS & HF-rTMS across various domains. A potential mechanistic explanation for this is that LF-rTMS (≤1 Hz) modulates neural hyperactivity in lesioned regions via inhibitory neuromodulation, whereas HF-rTMS (≥5 Hz) facilitates language recovery through excitatory stimulation of contralateral homologous areas (17, 58). Consistent with the hemispheric competition model, simultaneous bilateral stimulation may promote more effective recovery in patients (59). Regarding the alleviation of global aphasia severity, twice-daily low-frequency rTMS (2LF-rTMS) treatment demonstrated superior efficacy. This observation is supported by evidence indicating that high-dose interventions administered over short durations enhance speech therapy outcomes (60), potentially reflecting an underlying dose–response relationship (17). However, the optimal extent and tolerance limits are still unknown. For the improvement of repetition abilities, iTBS showed significant efficacy, corroborating findings by Bai et al. (61). We hypothesize that this effect may stem from iTBS stimulation of the left hemisphere of Broca’s area leading to increased activation in the superior frontal gyrus within the dominant frontal lobe (61). Furthermore, the neurophysiological effects of iTBS exhibit a dynamic temporal evolution, shifting from local activation at the stimulation site to the engagement of distal regions within the fronto-limbic network (62). Conversely, cTBS proved particularly effective for treating naming deficits, consistent with the results reported by Harvey et al. (63). This is likely attributable to cTBS being an inhibitory rTMS paradigm capable of inducing robust modulation of cortical excitability within a significantly shorter timeframe (<1 min) than required by conventional LF-rTMS to achieve comparable effects (64), additionally, while environmental noise levels can impact naming performance (65, 66), cTBS may enhance naming abilities in aphasia by improving the signal-to-noise ratio at the stage of lexical-to-phonological mapping (65, 67). Crucially, our study found that LF-rTMS demonstrated superior outcomes compared to HF-rTMS across all assessed aspects, a finding consistent with other research (68). This conclusion is further substantiated by a recent meta-analysis (69), which directly compared the efficacy of high- and low-frequency rTMS for aphasia treatment and concluded that low-frequency stimulation yields more significant therapeutic effects, demonstrating significant concordance with the present results.

Regarding the selection of stimulation sites, the POp and the PTr subserve functionally dissociable roles in phonological and semantic processing, respectively. Specifically, the PTr is primarily implicated in processing word meaning, whereas the POp is more critically involved in processing the sound patterns of words (70). The results of the present study demonstrated superior efficacy for POp stimulation compared to PTr stimulation across all language domains assessed. This finding aligns with the results reported by Wang et al. (14). The results of the present study demonstrated superior efficacy for POp stimulation compared to PTr stimulation across all language domains assessed. This finding aligns with the results reported by Naeser et al. (71). One potential explanation for the observed superiority of POp stimulation may relate to functional lateralization patterns. A previous study (5) reported no functional relationship between the right PTr and the left PTr. However, it found that the right POp demonstrated functional homology to the left POp. This right hemisphere functional homology for the POp might contribute to the enhanced efficacy observed when stimulating this site. Nevertheless, it is important to acknowledge potential limitations in our study that could have influenced the results. These include variability in the types, quantity, and methodological quality of the included literature, as well as the possibility of inaccuracies in stimulation site localization within some studies. Furthermore, the apparent discrepancy with Naeser’s et al. (71) findings warrants consideration. Their investigation focused specifically on the effect of stimulation sites on naming performance and did not evaluate outcomes across other language domains. This narrower scope may account for the inconsistency with our results, which encompassed a broader range of language functions.

Regarding the choice of localization modality, neuronavigation systems facilitate the identification of optimal target structures for rTMS. Conventional approaches utilizing the 10–20 EEG system are susceptible to inaccurate coil positioning within the target area (72). Neuronavigation addresses this limitation by providing real-time visualization and feedback of coil position, enabling more precise localization and stabilization of stimulus delivery. In the present study, AL demonstrated greater efficacy than alternative methods in patients with aphasia. This finding contrasts with the results reported by Bashir et al. (73); several factors may account for this discrepancy. First, the characteristics of the included literature (e.g., quality and quantity) in our meta-analysis may have influenced the outcome. Furthermore, a critical methodological difference exists: Bashir’s et al. study investigated healthy individuals, whereas our analysis focused specifically on patients with post-stroke aphasia. This fundamental difference in study populations likely contributed significantly to the observed inconsistency in results.

### 4.1 Limitations

There are still some limitations of this study (1): the small number of studies included in some interventions, as well as the small number of interventions in studies, which may affect the reliability of the results; future research should focus on expanding sample sizes for these specific interventions and encouraging the reporting of comparative studies of multiple interventions (2). The results of the risk of bias assessment showed that the overall quality of the included studies was low. Key methodological limitations included unreported allocation concealment and blinding procedures in several studies. Failure to implement allocation concealment compromises randomization and may introduce selection bias. Lack of blinding can lead to detection bias or performance bias due to subjective influences during data collection or intervention delivery. Additionally, incomplete outcome data, notably within the spontaneous speech domain, resulted in reduced sample sizes for some analyses. These limitations may compromise the reliability and validity of our findings. It is recommended that future studies strictly adhere to reporting specifications and report in detail on randomization, allocation concealment, blinding, and missing data handling methods (3). The study differed between patients in terms of the type of aphasia, duration, frequency of treatment, intensity, site of intervention, and method of assessment, which may have led to findings of low-quality evidence. Future studies should aim to standardize key operational parameters, clarify report positioning methods, and stratify analyses or design trials with greater homogeneity based on core patient characteristics (4). The included studies utilized diverse assessment scales, introducing significant heterogeneity that limits the comparability and interpretation of the pooled results. Although standardized mean differences (SMDs) were used to combine outcomes, fundamental differences in scale reliability, sensitivity, measurement range, and potential cultural bias (e.g., omission of culturally specific symptoms when Western scales are directly translated into Chinese) may contribute to measurement bias. Consequently, the findings should be interpreted with caution. To enhance future research validity, developing standardized assessment guidelines and adopting established, high-reliability consensus scales (e.g., Western aphasia battery, WAB) is strongly recommended. Furthermore, we were able to partially address this heterogeneity by conducting a subgroup analysis based on scale type (5). Most of the literature is from China, which may generate language bias. Study design, intervention implementation, and outcome measures may be influenced by specific linguistic/cultural contexts. Thus, extrapolation of the current findings is significantly limited and may not adequately represent the diversity of the global aphasic population. Therefore, the impact of linguistic and cultural factors on intervention and assessment needs to be considered in future research.

## 5 Conclusion

In summary, this study evaluated the efficacy of different types of transcranial magnetic stimulation for post-stroke aphasia using the NMA system. It clarified the ranking of the advantages and disadvantages of each treatment option across various language domains. LF-TMS & HF-TMS demonstrated superior efficacy across multiple therapeutic indices, suggesting LF-TMS & HF-TMS as the most preferred interventions to be used in the clinic treatment of post-stroke aphasia. iTBS was particularly effective for improving comprehension and repetition, supporting its recommendation as a targeted therapeutic approach for patients with deficits in these domains. In terms of naming, cTBS performs well, but there is a lack of studies based on cTBS, so further clinical validation of the efficacy of cTBS is needed. When choosing IFG as the stimulation site, POp is the optimal choice. For targeting method, the 10–20 EEG system positioning currently represents the most evidence-supported approach, though this conclusion requires further validation through prospective comparative studies.

The conclusions of this review should be interpreted with caution due to the overall high risk of bias identified in the included studies and the significant heterogeneity introduced by diverse assessment scales. Therefore, the findings are considered exploratory and hypothesis-generating, requiring further validation through future high-quality RCTs employing standardized outcome measures and robust methodologies.
